# Dietary supplementation with biogenic selenium nanoparticles alleviate oxidative stress-induced intestinal barrier dysfunction

**DOI:** 10.1038/s41538-022-00145-3

**Published:** 2022-06-23

**Authors:** Lei Qiao, Xinyi Zhang, Shanyao Pi, Jiajing Chang, Xina Dou, Shuqi Yan, Xiaofan Song, Yue Chen, Xiaonan Zeng, Lixu Zhu, Chunlan Xu

**Affiliations:** The Key Laboratory for Space Bioscience and Biotechnology, School of Life Sciences, Northwestern Polytechnical University, Xi’an, Shaanxi 710072 China

**Keywords:** Gastrointestinal diseases, Nutrition

## Abstract

Selenium (Se) is an essential micronutrient that promotes body health. Endemic Se deficiency is a major nutritional challenge worldwide. The low toxicity, high bioavailability, and unique properties of biogenic Se nanoparticles (SeNPs) allow them to be used as a therapeutic drug and Se nutritional supplement. This study was conducted to investigate the regulatory effects of dietary SeNPs supplementation on the oxidative stress-induced intestinal barrier dysfunction and its association with mitochondrial function and gut microbiota in mice. The effects of dietary SeNPs on intestinal barrier function and antioxidant capacity and its correlation with gut microbiota were further evaluated by a fecal microbiota transplantation experiment. The results showed that Se deficiency caused a redox imbalance, increased the levels of pro-inflammatory cytokines, altered the composition of the gut microbiota, and impaired mitochondrial structure and function, and intestinal barrier injury. Exogenous supplementation with biogenic SeNPs effectively alleviated diquat-induced intestinal barrier dysfunction by enhancing the antioxidant capacity, inhibiting the overproduction of reactive oxygen species (ROS), preventing the impairment of mitochondrial structure and function, regulating the immune response, maintaining intestinal microbiota homeostasis by regulating nuclear factor (erythroid-derived-2)-like 2 (Nrf2)-mediated NLR family pyrin domain containing 3 (NLRP3) signaling pathway. In addition, Se deficiency resulted in a gut microbiota phenotype that is more susceptible to diquat-induced intestinal barrier dysfunction. Supranutritional SeNPs intake can optimize the gut microbiota to protect against intestinal dysfunctions. This study demonstrates that dietary supplementation of SeNPs can prevent oxidative stress-induced intestinal barrier dysfunction through its regulation of mitochondria and gut microbiota.

## Introduction

Selenium (Se) as an essential micronutrient element plays a key role in anti-oxidation, anti-cancer, antiviral and immune responses. The human body relies on adequate dietary Se intake and this nutrient exerts its biological effects mostly through its incorporation into selenoproteins^1^. In humans, 25 selenoproteins have been identified^2,3^. Most selenoproteins, such as glutathione peroxidase (GPx) and thioredoxin reductase (TrxR), have redox activity and are widely distributed in different tissues and organs to perform multiple functions. GPx use glutathione (GSH) as a reducing agent to reduce hydroperoxide and hydrogen peroxide, thereby acting as an antioxidant. TrxR catalyzes the reduction of disulfides at the active site of thioredoxin (Trx) to maintain cell redox homeostasis^4^. Selenoproteins F, K, and S are involved in the redox regulation of endoplasmic reticulum, protein folding and cellular calcium homeostasis to varying degrees^5^.

Se gets into the food chain through plants which take it up from the soil. However, the distribution of Se on the earth is very uneven, daily dietary Se intake in humans shows high variability globally^6^. Se deficiency is a causative factor in various diseases, including cardiomyopathy, Keshan disease, intestinal disease, and also epilepsy and age-associated neurological disorders, and has become a common public nutrition problem in Europe, the Middle East, as well as some Asian countries^7–9^. Therefore, understanding the mechanisms of effects of Se deficiency on human health is critical for solving health problems associated with Se intake imbalance^10^. The gastrointestinal tract is a highly complex organ and a key interaction site for epithelial cells, immune cells, luminal contents, and microbiota. An intact intestinal barrier is of great importance to human health. Due to continuous exposure to exogenous factors, the intestine is very susceptible to oxidative stress. Maintenance of proper function and balance between intestinal cell populations is a tightly controlled process that relies heavily on proper redox homeostasis^11,12^. Thus, disruption of oxidative balance is believed to contribute to various types of intestinal injuries and diseases. Mitochondria are important sites for various cellular processes in eukaryotic cells. Therefore, well-controlled mitochondrial number, quality, and function play a crucial role in maintaining the physiological state of epithelial cells^13–15^. Most oxidative reactions occur in mitochondria, which are organelles that contain enzymes with Se as a cofactor^16^. Se has been shown to have antioxidant and protective effects in cells by modulating mitochondrial function and inducing mitochondrial biogenesis. Yang et al.^17^ found that Se supplementation alleviated the damage of mitochondrial structure caused by hyperglycemic ischemia, and further promoted mitochondrial biogenesis. Supplementation with Se significantly increased the levels of mitochondrial biogenesis markers and mitochondrial proteins and increased the activity of the electron transport chain complex^18^. In general, the positive effect of Se is mediated by lowering reactive oxygen species (ROS) production/accumulation and preserving the mitochondrial membrane potential (MMP) and mitochondrial functional performance^19^.

Recently, the influence of Se on the gut has attracted increasing attention. Human studies have routinely demonstrated that significant reductions in the serum Se levels in ulcerative colitis (UC) and Crohn’s disease (CD) patients and serum Se levels have consistently been found to be inversely correlated with UC severity, inflammatory bowel disease (IBD) duration, and CD activity index. Se has been proposed as a noninvasive biomarker for IBD activity and severity^11^. In nature, Se exists mainly in the inorganic state, with high toxicity and low bioavailability. Se nanoparticles (SeNPs) with unique biological properties have been suggested as a safer and more effective platform for the delivery of Se for biological needs. SeNPs are involved in the synthesis of various antioxidant proteins, such as GPx and TrxR, and thus have antagonistic effects on many diseases caused by oxidative stress, such as arthritis, tumors, and neurocardiovascular diseases^20,21^. In our previous study, we found that SeNPs synthesized by *Lactobacillus casei* ATCC 393 (*L. casei* ATCC 393) effectively alleviated the intestinal epithelial barrier dysfunction caused by diquat in vitro^22,23^. In addition, other studies have shown that dietary Se supplementation affects the composition and abundance of gut microbiota, which in turn will affect the bioavailability of Se and selenoprotein expression in mice^24^.

The above studies suggest that there may be a potential interaction between Se intake, mitochondria, and gut microbiota. Based on this suggestion, we hypothesized that the benefits of dietary SeNPs on intestinal health are not only related to the regulation of redox balance, but may also be related to their impact on the gut microbiota. Therefore, this study compared the effects of dietary supplementation with different concentrations of SeNPs (0.0-Se, 0.3-Se, and 0.6-Se mg/kg) on the intestinal barrier dysfunction, mitochondria structure and function, and gut microbiota in mice. Furthermore, through fecal microbiota transplantation (FMT) to bypass the influence of Se itself, we investigated whether the effects of dietary SeNPs supplementation on the intestinal barrier dysfunction, antioxidant capacity, and immune responses are associated with its modulation of the gut microbiota.

## Results

### Effects of different dietary SeNPs supplementation on growth performance and Se content of mice

**Fig. 1:**
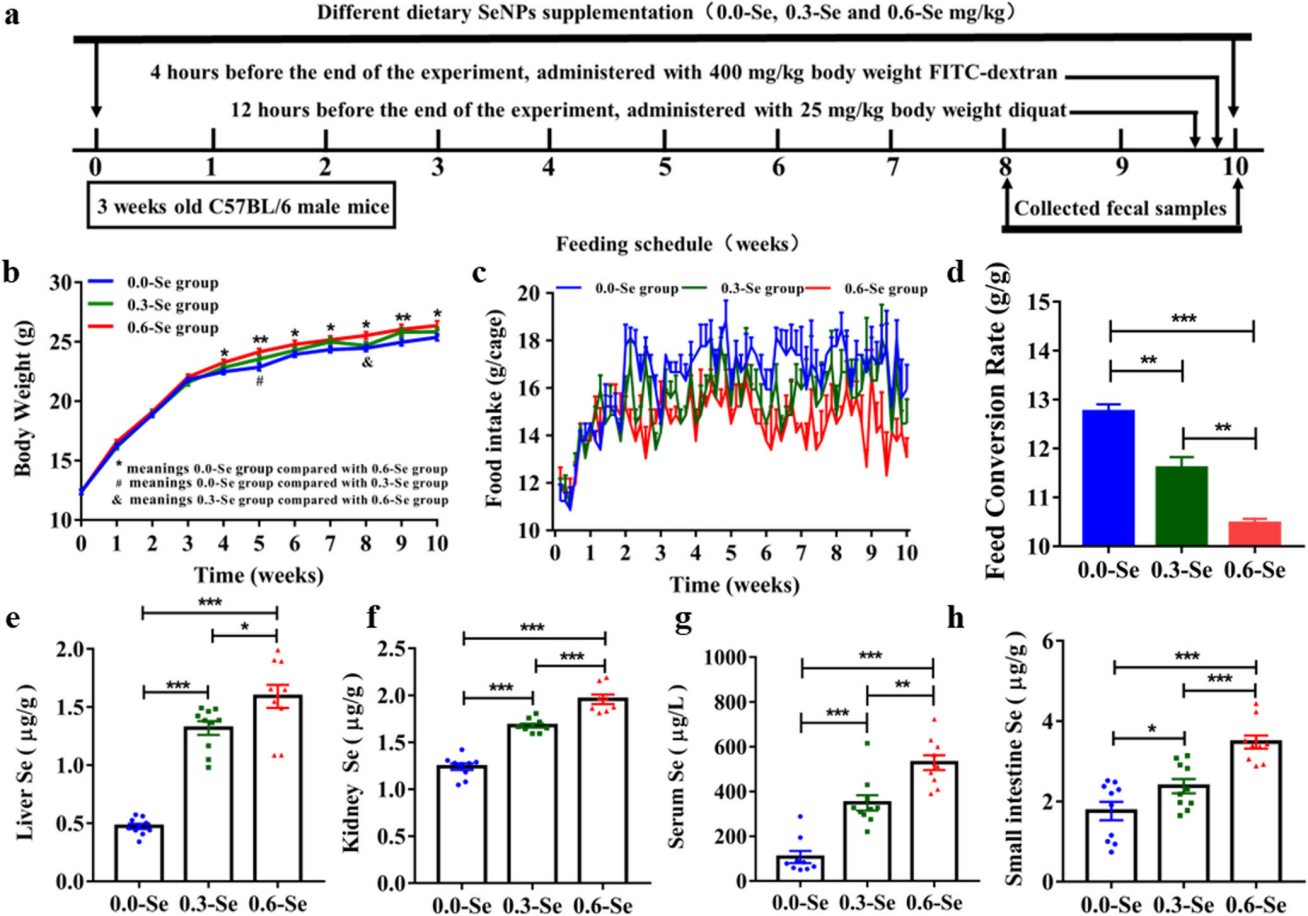
Effects of different dietary SeNPs supplementation on growth performance and Se content of mice. **a** Schematic diagram of experiment. **b** Body weight and **c** food intake was recorded throughout the 10-week period experiment. **d** Feed conversion rate. **e**–**h** The Se content in the liver, kidney, serum, and small intestine, respectively, (*n* = 10). Data are expressed as mean ± SEM. ^*^P < 0.05; ^**^P < 0.01; ^***^P < 0.001.

The 10-week supplementation period with different SeNPs-containing diets (0.0-Se, 0.3-Se, and 0.6-Se mg/kg) resulted in marked variations in body weight, food intake and feed conversion rate [FCR = (final weight-initial weight)/feed intake, g/g] of the mice (Fig. 1b–d). Final body weight was higher in mice fed the 0.6-Se diet than in those fed the 0.0-Se diet, but there were no significant differences with the group fed the 0.3-Se diet. The final feed conversion rate of mice in the 0.3-Se and 0.6-Se supplementation groups were significantly lower compared with that of mice in the 0.0-Se group. However, compared with the 0.3-Se group mice, the feed conversion rate of the 0.6-Se group mice was the lowest. This result indicated that the feed conversion efficiency of the 0.6-Se group is the highest. The content of Se in organs, serum, and feces after ten weeks of supplementation was dietary Se concentration-dependent (Fig. 1e–h and Supplementary Fig. 1). Se concentration in liver, kidney, serum, and small intestine of the 0.0-Se group was significantly lower compared with that in the 0.3-Se and 0.6-Se groups, and the Se content in the 0.6-Se group was higher than that in the 0.3-Se group. This implied decreased Se availability in mice in the 0.0-Se and 0.3-Se groups. Se exerts its physiological functions in nutrition, metabolism and immunity largely through selenoproteins. The effects of different SeNPs-containing diets on the expression of selenoprotein genes in the liver and jejunum of mice are shown in Supplementary Fig. 2. Dietary SeNPs supplementation upregulated the expression of most selenoprotein genes in liver (except *GPX1*, *GPX4*, *SELENOI*, *SELENOO,* and *SELENOW*) in a dose-dependent manner. Similarly, except for the extremely low expression of the DIO enzyme family and *SELENOV* in the jejunum, the mRNA expression of most other selenoproteins also increased with the increase of the Se content. Overall, these results supported the notion that the different dietary SeNPs supplementation had an effect on the expression levels of selenoprotein genes in these mice.

### Effects of different dietary SeNPs supplementation on the intestinal barrier function in mice exposed to diquat

**Fig. 2:**
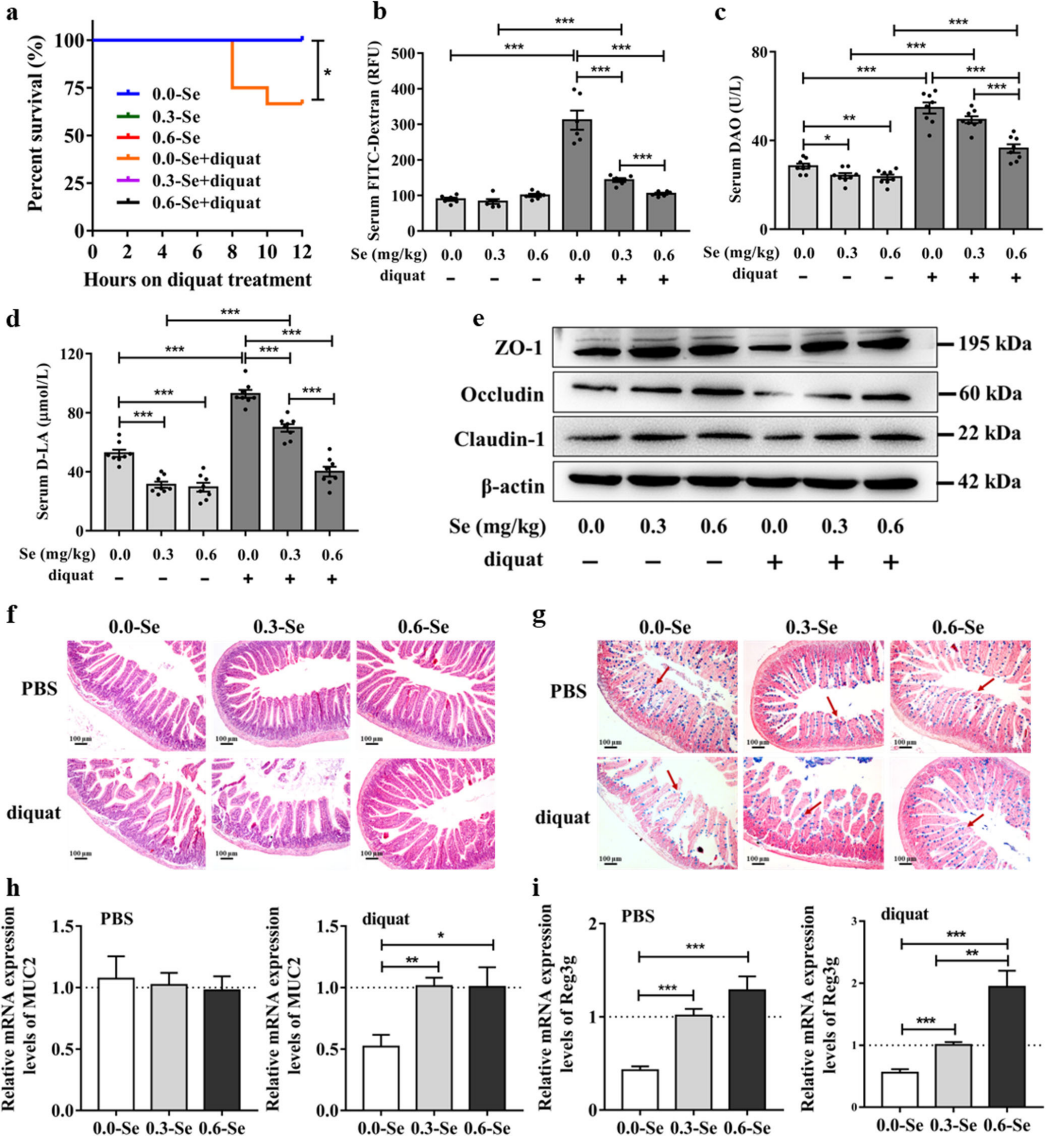
Effects of different dietary SeNPs supplementation on the intestinal barrier in mice exposed to diquat. **a** Survival rate of mice over a 12-h period during diquat treatment. **b**–**d** Biomarkers related to intestinal barrier function damage including FITC-dextran (**b**) (*n* = 6), DAO (**c**) (*n* = 8), D-LA (**d**) (*n* = 8). **e** The expression levels of tight junction proteins (ZO-1, occludin, claudin 1) detected by Western blot analysis (*n* = 3). **f** Jejunum morphology was observed by H&E staining (*n* = 4). **g** Goblet cells in the jejunum were observed by AB-PAS staining. **h** mRNA expression levels of *MUC2* in the jejunum (*n* = 6). i mRNA expression levels of *REG3G* in the jejunum (*n* = 6). Data are expressed as the fold change versus the 0.3-Se group or 0.3-Se + diquat group (set to 1). Data are expressed as mean ± SEM. ^*^P < 0.05; ^**^P < 0.01; ^***^P < 0.001.

The survival rates of mice exposed to diquat in 0.0-Se, 0.3-Se, and 0.6-Se groups were 66.67%, 100%, and 100%, respectively (Fig. 2a). The detection of biomarkers related to intestinal barrier dysfunction, including FITC-dextran, Diamine oxidase (DAO), D-lactic acid (D-LA), and tight junction proteins (ZO-1, occludin, and claudin1) expression (Fig. 2b–e and Supplementary Fig. 3a–c) revealed that in the normal control group, the activity of DAO and the level of D-LA in the Se-deficient group were significantly higher than those in the dietary SeNPs supplementation group. Conversely, significantly decreased levels of expression of tight junction proteins were detected in mice fed with 0.0-Se diet compared with those in the dietary SeNPs supplementation group. Moreover, compared with the normal control group, the activity of DAO, the levels of D-LA and FITC-dextran in the jejunum of diquat-exposed mice were significantly increased, and the expression of tight junction proteins was significantly decreased. However, dietary biogenic SeNPs supplementation (especially at supernutritional level, 0.6-Se mg/kg) effectively alleviated the intestinal barrier injury caused by diquat. To further assess the jejunum injury, histology was performed on jejunal tissue sections and the villus height and the number of goblet cells in hematoxylin and eosin (H&E) and alcian blue (AB)-periodic acid schiff (PAS) stained tissue sections were determined (Fig. 2f and g). Also, compared with the normal control group, the jejunal villi were disorderly arranged, the villus height was shortened (Supplementary Fig. 3d), and the number of goblet cells was decreased in mice exposed to diquat. However, compared with the diquat-induced oxidative stress model group, dietary 0.6-Se mg/kg supplementation significantly increased the height of the villi and the number of goblet cells (Supplementary Fig. 3e). The mRNA expression levels of mucin 2 (*MUC2*) and regenerating family member 3gamma (*REG3G*) in jejunum were detected by qPCR analysis. As shown in Fig. 2h and i, in normal control group, different dietary SeNPs supplements did not affect the expression of *MUC2*, but compared with the 0.3-Se and 0.6-Se groups, the expression of *REG3G* in the 0.0-S group was significantly reduced. In the diquat-induced oxidative stress model group, dietary SeNPs supplementation upregulated the expression of *MUC2* and *REG3G* in the jejunum, and had a dose-dependent effect. This indicates that biogenic SeNPs effectively alleviated the diquat-induced intestinal barrier dysfunction.

### Effects of different dietary SeNPs supplementation on the antioxidant capacity and immune responses

**Fig. 3:**
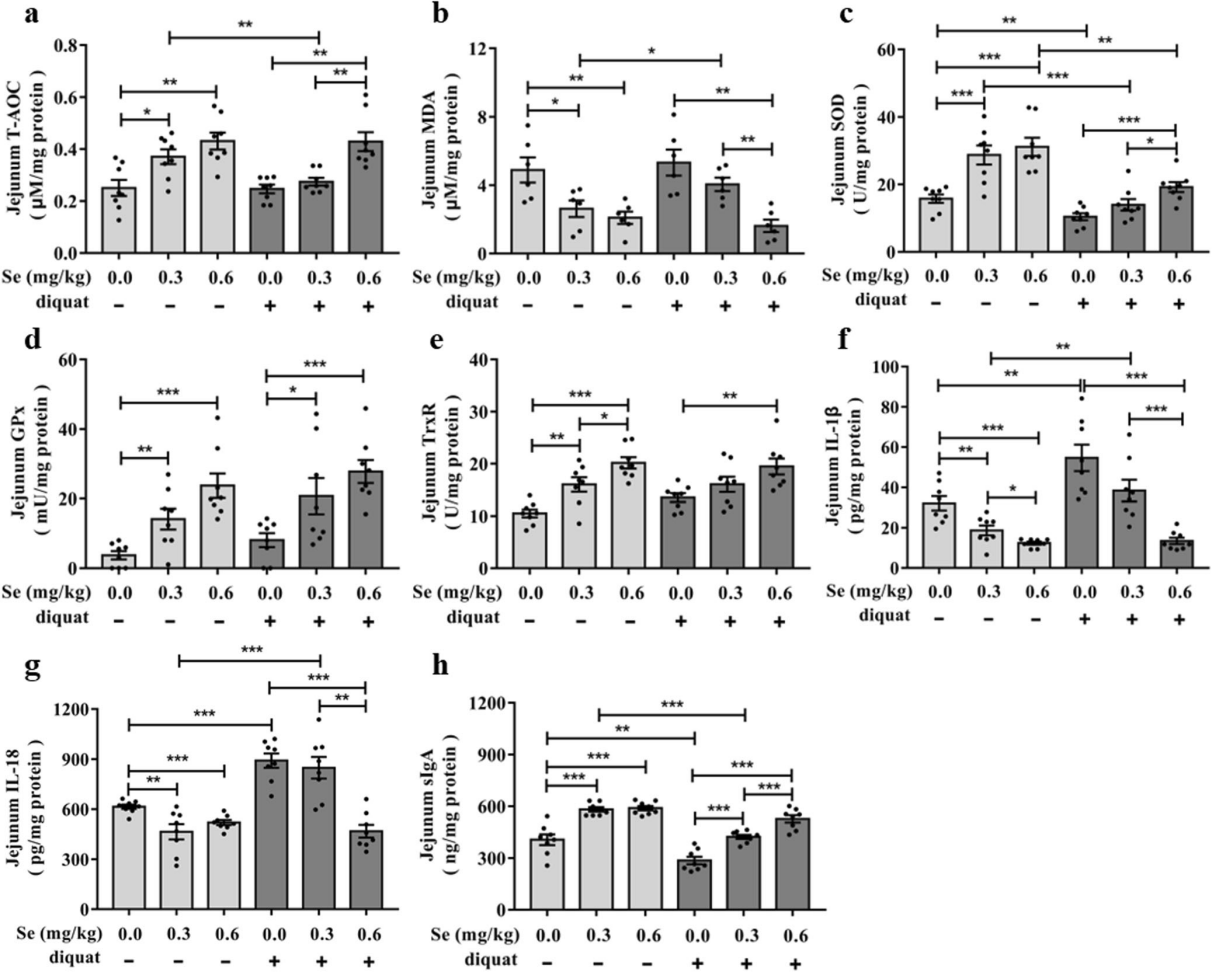
Effects of different dietary SeNPs supplementation on the antioxidant capacity and immune response in diquat-exposed mice. **a**–**e** Oxidative stress response including T-AOC (**a**), MDA (**b**), SOD (**c**), GPx (**d**), TrxR (**e**) (*n* = 8). **f** Level of IL-1β in the jejunum of mice (*n* = 8). **g** Level of IL-18 in the jejunum of mice (*n* = 8). **h** Level of sIgA in the jejunum of mice (*n* = 8). Data are expressed as mean ± SEM. ^*^P < 0.05; ^**^P < 0.01; ^***^P < 0.001.

As expected, decreased Se availability weakened the antioxidant capacity of jejunum by decreasing total antioxidant capacity (T-AOC), superoxide dismutase (SOD), GPx and TrxR levels, and increased the level of the lipid peroxidation product, malondialdehyde (MDA). Moreover, diquat exposure significantly aggravated the decline in the antioxidant capacity of the jejunum. Noteworthy, the addition of 0.6-Se mg/kg of SeNPs to the diet can effectively alleviate the decrease in the antioxidant capacity of the mice jejunum caused by diquat (Fig. 3a–e). The levels of biomarkers related to immune responses in the serum and jejunum were also markedly altered by different dietary SeNPs supplementation from diquat-exposed and normal control groups (Fig. 3f–h and Supplementary Fig. 4). The results demonstrated that Se deficiency can lead to an increase in the levels of interleukin-1β (IL-1β) and interleukin-18 (IL-18), and a decrease in the level of secretory immunoglobulin A (sIgA). Moreover, the 0.6-Se group exposed to diquat had significantly lower levels of IL-1β and IL-18 and higher level of sIgA compared with diquat-exposed mice fed either 0.0 or 0.3 mg/kg Se diets. Notably, mice in the 0.3-Se diet were not significantly different from those in the 0.0-Se group. Thus, our results showed that 0.6 mg/kg SeNPs supplementation can improve the antioxidant capacity and relieve intestinal inflammation in diquat-exposed mice.

### Dietary SeNPs supplementation effectively alleviated diquat-induced intestinal mitochondrial dysfunction

**Fig. 4:**
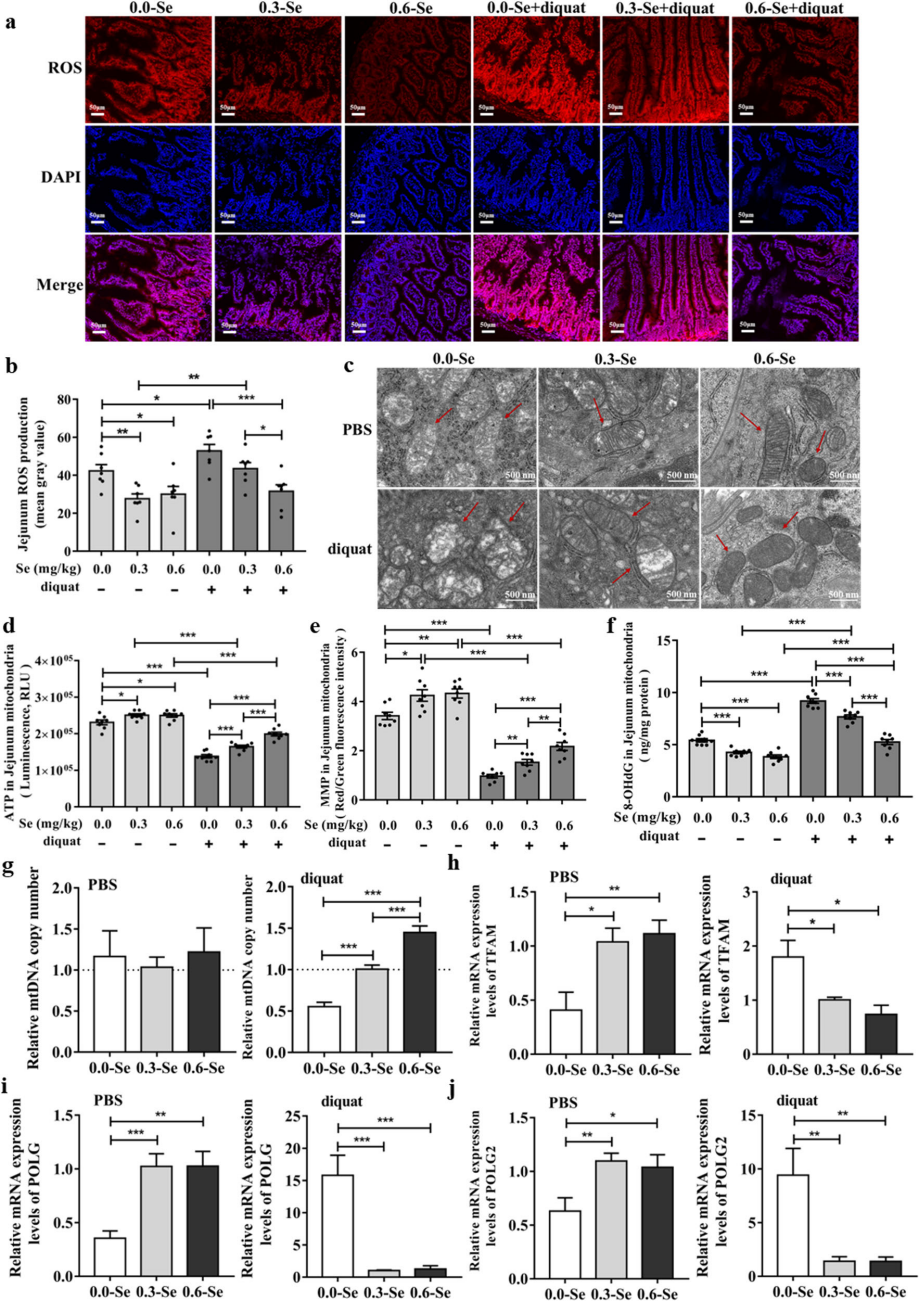
Effects of different dietary SeNPs supplementation on jejunal mitochondrial dysfunction in diquat-exposed mice. **a**, **b** ROS production was determined by DHE staining (*n* = 6). **c** Mitochondrial ultrastructure was observed by TEM. **d** Mitochondrial ATP levels in the jejunal mitochondria (*n* = 8). **e** MMP was measured by JC-1 staining in the jejunal mitochondria (*n* = 8). **f** Levels of 8-OHdG in the jejunal mitochondria (*n* = 8). **g** The mtDNA copy number was determined by qPCR analysis (*n* = 6). **h** mRNA expression level of *TFAM* in the jejunum (*n* = 6). **i** mRNA expression level of *POLG* in the jejunum (*n* = 6). **j** mRNA expression level of *POLG2* in the jejunum (*n* = 6). Data are expressed as the fold change versus the 0.3-Se group or 0.3-Se + diquat group (set to 1). Data are expressed as mean ± SEM. ^*^P < 0.05; ^**^P < 0.01; ^***^P < 0.001.

Se deficiency caused ROS overproduction compared with the 0.3-Se and 0.6-Se groups, and the 0.6-Se group mice exposed to diquat had significantly decreased the levels of ROS in jejunal tissues compared with diquat-exposed mice fed either 0.0 or 0.3 mg/kg Se diets (Fig. 4a and b). As the key organelle for energy metabolism and ROS production, mitochondria are closely related to the redox state of cells. Oxidative stress from mitochondria may be a pathophysiological signal of intestinal barrier dysfunction. Thus, mitochondrial ultrastructure was examined by TEM, and the levels of 8-hydroxy-2’-deoxyguanosine (8-OHdG), ATP, and MMP in jejunal mitochondria were determined. In normal control, compared with the 0.3-Se and 0.6-Se groups, the ultrastructure of mitochondria in 0.0-Se group showed apparent changes, such as the swelling was obvious, the mitochondrial cristae were vague, and the density of matrix was low, less mitochondrial membrane was not intact. In addition, the highest degree of mitochondrial ultrastructural destruction (vacuoles were present, and most mitochondrial membranes were incomplete) was observed in the mitochondria of diquat-exposed mice from the 0.0-Se mg/kg diet group. However, the 0.6-Se group mice were significantly alleviated by the diquat-induced destruction of mitochondria, their mitochondria had a column or mesh shape, the mitochondrial cristae were clear, the density of matrix was normal, and the mitochondrial membrane was intact (Fig. 4c). The destruction of mitochondrial structure also affects mitochondrial function. Compared with the SeNPs supplementation group, the levels of ATP and MMP in the mitochondria of the Se-deficient mice from the normal control decreased significantly. Moreover, compared with the normal control, diquat-exposed further reduced the levels of ATP and MMP, and dietary SeNPs (0.6-Se mg/kg) supplementation effectively alleviated diquat-induced intestinal mitochondrial dysfunction (Fig. 4d and e). The level of 8-OHdG (a biomarker of DNA oxidative damage) in the 0.0-Se group mice was significantly higher than that in the 0.3-Se and 0.6-Se group mice relative to the normal control. However, the 0.6-Se mg/kg SeNPs supplementation was able to significantly protect mitochondrial DNA from diquat-induced oxidative stress damage (Fig. 4f). As shown in Fig. 4g, different dietary SeNP supplements did not affect the mtDNA copy number in the normal control group. In the diquat-exposed group, dietary SeNPs supplementation increased the mtDNA copy number in the jejunum in a dose-dependent manner. Various factors involved in the regulation and repair of mammalian mtDNA replication, including mitochondrial transcription factor A (*TFAM*), DNA Polymerase gamma (*POLG*), and DNA Polymerase gamma 2 (*POLG2*), play an important role in the repair of mitochondrial oxidative stress damage (Fig. 4h–j). Notably, different dietary SeNPs supplementation on the expression of genes related to mitochondrial biogenesis in the normal control group was opposite to that in the diquat-exposed group, specifically, the expression of *TFAM*, *POLG*, and *POLG2* in the chronic Se-deficient diet group was lower than that in the 0.3-Se and 0.6-Se groups. However, in the diquat-exposed group (acute stress), the expression of genes related to mitochondrial biogenesis in the 0.0-Se group was higher than that in the 0.3-Se and 0.6-Se groups.

### Dietary SeNPs supplementation altered the composition and metabolism of gut microbiota in mice

**Fig. 5:**
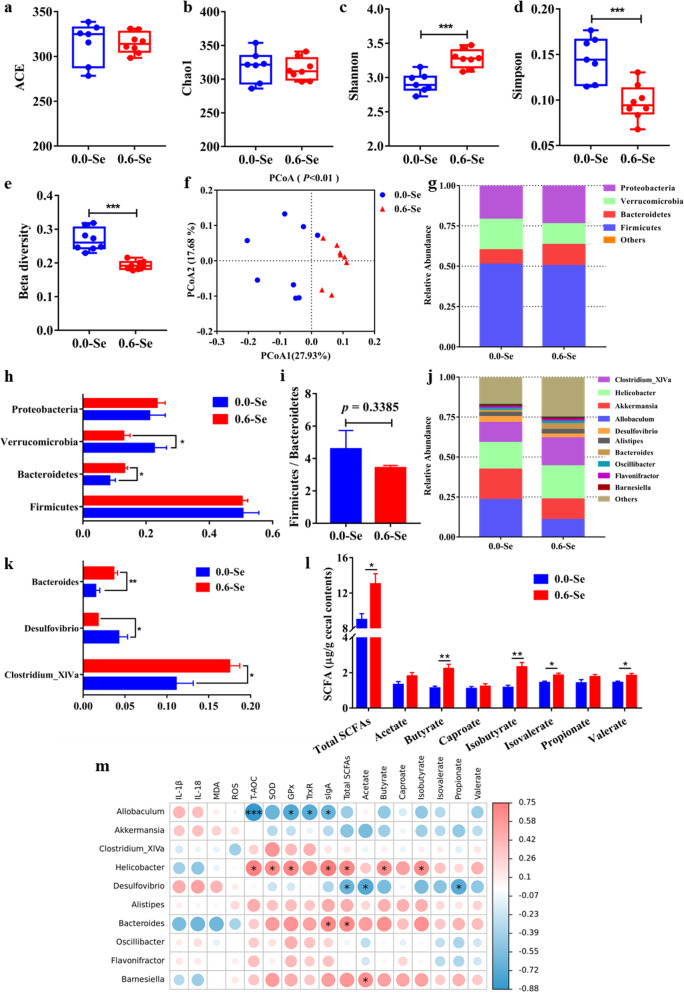
Effects of different dietary SeNPs supplementation on gut microbiota and SCFAs levels of mice. **a**–**d** Alpha diversity index (*n* ≥ 7). **e** Beta diversity index (*n* ≥ 7). **f** PCoA (*n* ≥ 7). **g** The relative abundance composition of fecal microbiota at the phylum level (*n* ≥ 7). **h** Differences in fecal microbiota between the phylum level (*n* ≥ 7). **i** Ratio of *Firmicutes* to *Bacteroides* (*n* ≥ 7). **j** The relative abundance composition of fecal microbiota at the genus level (*n* ≥ 7). **k** Differences in fecal microbiota between the genus level (*n* ≥ 7). **l** SCFAs levels in the cecal contents of mice (*n* = 5). **m** Heatmap of Spearman’s correlation between the abundance of gut microbiota and the intestinal barrier dysfunction-related biochemical indexes. Data are expressed as mean ± SEM. ^*^P < 0.05; ^**^P < 0.01; ^***^P < 0.001.

Numerous studies have shown that dietary changes will alter the composition of the gut microbiota. Indeed, in this study, the findings of significant differences in body weight, food intake, and feed conversion rate between the 0.0-Se group and the 0.6-Se group suggest that the dietary SeNPs may have altered the composition of the gut microbiota. As shown in Fig. 5a and b, the ACE and Chao1 indices were used to measure the community richness of gut microbiota. However, supplementation with different concentrations of SeNPs did not significantly alter the overall richness of the gut microbiota. The Shannon and Simpson diversity indices were used to evaluate the diversity of gut microbiota (Fig. 5c and d). Compared to the 0.0-Se group, the Shannon index was increased and the Simpson index was decreased in the 0.6-Se group which indicates that dietary SeNPs supplementation can significantly alter the diversity of the gut microbiota of mice. Beta diversity is shown in Fig. 5e, the samples in the 0.6-Se group have a significantly lower level of dispersion than those in the 0.0-Se group. Additional principal coordinates analysis (PCoA) found that the gut microbiota of mice in different SeNPs supplementation groups showed obvious clustering and the 0.6-Se group was clearly separated from the 0.0-Se group, indicating that SeNPs significantly changed the composition of the gut microbiota (Fig. 5f). Biogenic SeNPs induced modulation of the gut microbiota structure at the phylum level, resulting in an enhanced abundance of *Bacteroidetes* and a reduced abundance of *Verrucomicrobia* (Fig. 5g and h). The Firmicutes/Bacteroidetes (F/B) ratio has been suggested as an important index of the health of the gut microbiota. A decreasing trend in the F/B ratio was observed in the 0.6-Se group compared with the 0.0-Se group (*P* = 0.3385, Fig. 5i). At the genus level, significant increases in the levels of *Bacteroides* and *Clostridium_XlVa* and decreases in the level of *Desulfovibrio* were detected in mice fed with the 0.6-Se diet compared with results for mice fed with the 0.0-Se diet (Fig. 5j and k). Similar alterations in the levels of SCFAs were also observed in the cecal contents of mice fed with different SeNPs supplementation doses. Compared with 0.0-Se group, supranutritional Se increased the content of total SCFAs, butyrate, isobutyrate, valerate, and isovalerate (Fig. 5l). Subsequently, using PICRUSt2 software and the KEGG database, we analyzed the difference and changes in metabolic pathways of functional genes of the gut microbiota. The enriched functional categories in the 0.6-Se group included “Human Diseases” at the KEGG level 1 and “Digestive system” at the KEGG level 2 (Supplementary Figs. 5 and 6).

A heatmap of Spearman correlation between the altered genera and intestinal barrier dysfunction-related biochemical indexes (the levels of inflammatory cytokines and antioxidant capacity in jejunum) was generated to identify the potential correlation between Se-induced gut microbiota changes and intestinal barrier dysfunction. As shown in Fig. 5m, *Allobaculum* was negatively correlated with the antioxidant capacity including T-AOC, GPx, and TrxR, while *Helicobacter* was positively correlated with the activities of T-AOC, GPx, and TrxR. Moreover, *Helicobacter* and *Bacteroides* were positively correlated with the level of sIgA, but negatively correlated with *Allobaculum*. In addition, for SCFAs, *Helicobacter* was positively correlated with total SCFAs, butyrate, and isobutyrate, *Bacteroides* was positively correlated with total SCFAs, and *Desulfovibrio* was negatively correlated with total SCFAs, acetate, and propionate. It’s worth noting that the abundance of *Desulfovibrio* significantly increased in the 0.0-Se group, and the abundance of *Bacteroides* was significantly decreased, illustrating that Se deficiency reduces the abundance of SCFAs-producing bacteria and increases the abundance of pathogenic bacteria that cause oxidative stress.

### The Nrf2 signaling pathway inhibits diquat-induced NLRP3 inflammasome activation in the jejunum

**Fig. 6:**
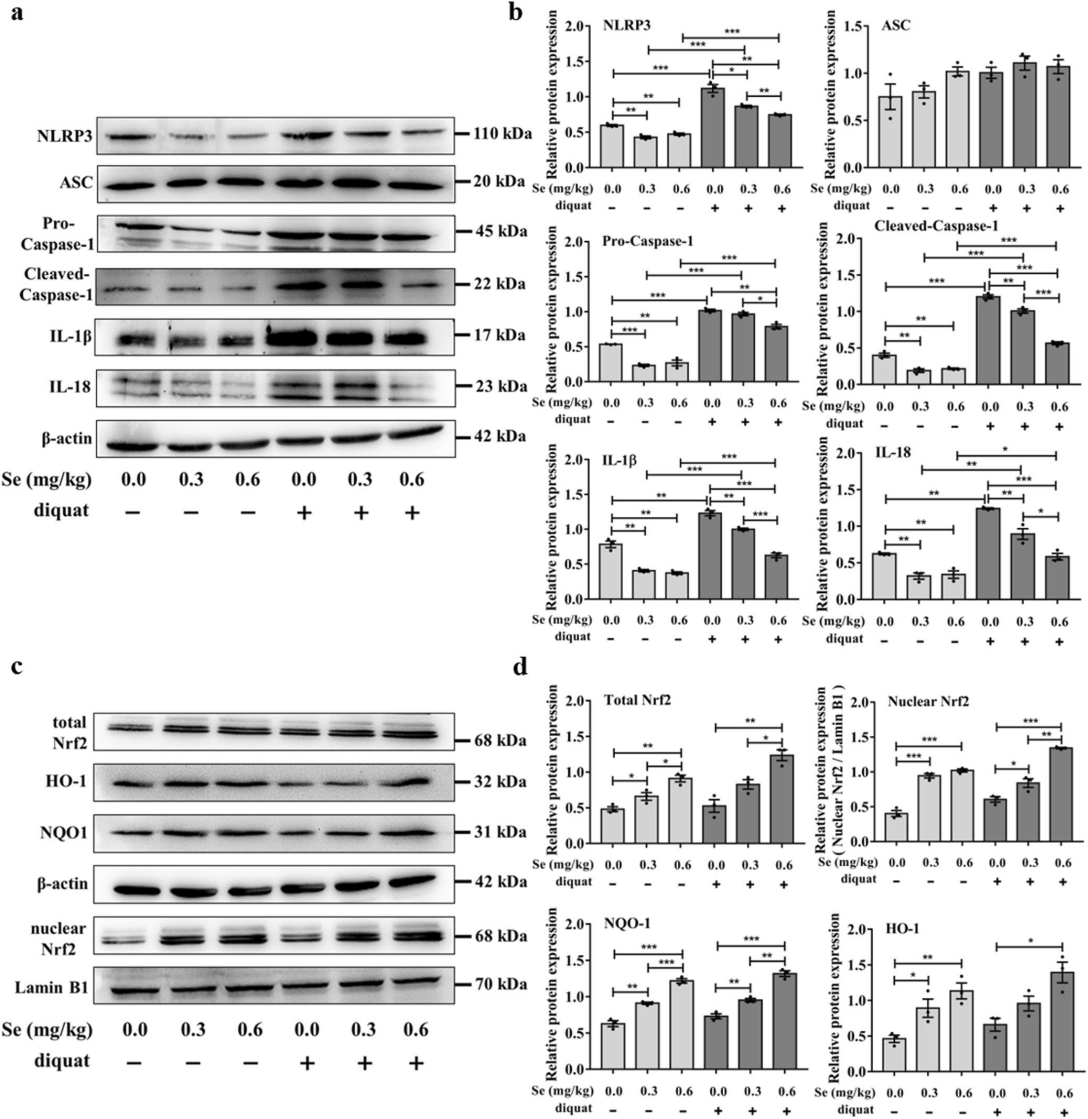
The Nrf2 signaling pathway inhibits the diquat-induced NLRP3 inflammasome activation in the jejunum. **a** The expressions of the NLRP3 inflammatory proteins were measured by Western blot analysis (*n* = 3). **b** Quantitative analysis of the protein expression levels of NLRP3, ASC, pro-caspase 1, cleaved-caspase 1, IL-1β, and IL-18. **c** Nrf2 activation and expression levels of its downstream proteins were measured by Western blot analysis (*n* = 3). **d** Quantitative analysis of the protein expression levels of total Nrf2, nuclear Nrf2, HO-1, and NQO-1. Data are expressed as mean ± SEM. ^*^P < 0.05; ^**^P < 0.01; ^***^P < 0.001.

To begin to understand the possible mechanisms by which dietary SeNPs may influence the responses to diquat exposure in this mice model, we decided to focus our research on the NLR family pyrin domain containing 3 (NLRP3) inflammasome signaling pathway. The reasons for this decision are that mitochondrial dysfunction plays a key role in activating the NLRP3 inflammasome, and ROS released by damaged mitochondria can promote its activation and the nuclear factor (erythroid-derived-2)-like 2 (Nrf2) transcription factor is a key player in cytoprotection and activated in stress conditions caused by ROS. Our results indicated that Se deficiency can lead to an increase in the level of the NLRP3 inflammasome and subsequent IL-1β and IL-18 expression, and diquat-exposed further increased its expression. However, the levels of the NLRP3 inflammasome and subsequent IL-1β and IL-18 expression showed decreasing trends along with the increasing levels of dietary Se in the jejunum of mice exposed to diquat (Fig. 6a and b). Nrf2 is a master regulator of the antioxidant response and has been implicated in a range of chronic diseases that are characteristically associated with oxidative stress. As shown in Fig. 6c and d, dietary SeNPs supplementation upregulated the expression of Nrf2 (total Nrf2 and nuclear Nrf2) and downstream antioxidant proteins NADPH dehydrogenase (NQO)-1 and heme oxygenase (HO)-1 in a dose-dependent manner, regardless of whether they were exposed to diquat or not.

### Effects of FMT on intestinal barrier in mice exposed to diquat

**Fig. 7:**
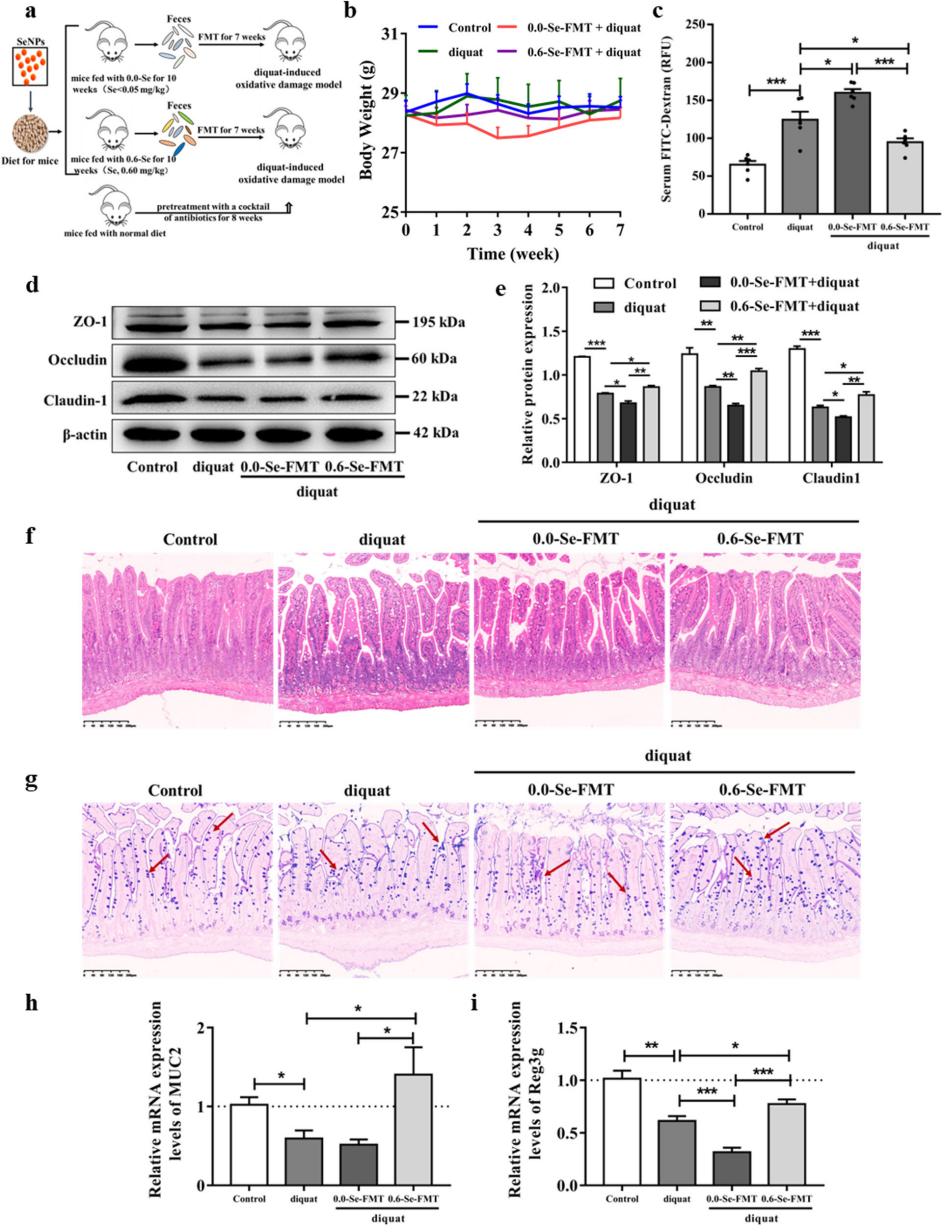
Effects of FMT on the intestinal barrier in mice exposed to diquat. **a** Schematic diagram of the FMT experiment. **b** Body weight during FMT. **c** FITC-dextran levels in the serum of mice. (*n* = 6). **d**, **e** The expression levels of tight junction proteins (ZO-1, occludin, claudin1) were measured by Western blot analysis (*n* = 3). **f** Jejunum morphology was observed by H&E staining (*n* = 4). **g** Goblet cells in the jejunum were observed by AB-PAS staining. **h** mRNA expression level of *MUC2* in the jejunum (*n* = 6). **i** mRNA expression level of *REG3G* in the jejunum (*n* = 6). Data are expressed as the fold change versus the 0.3-Se group or the 0.3-Se + diquat group (set to 1). Data are represented as mean ± SEM. ^*^P < 0.05; ^**^P < 0.01; ^***^P < 0.001.

We further investigated whether the effects of dietary SeNP supplementation on the intestinal barrier dysfunction and immune responses are associated with its modulation of the gut microbiota using the FMT method to bypass the influence of Se itself (Fig. 7a). As shown in Fig. 7b, the average body weight of the mice in the 0.0-Se-FMT group was slightly lower than that in the other SeNP supplementation groups, but there was no significant difference between the other groups before exposure to diquat. Mice colonized with the gut microbiota from Se-deficient donors showed more severe symptoms of diquat-induced intestinal barrier dysfunction compared with mice in the diquat-induced oxidative stress model group and 0.6-Se-FMT groups, as indicated by the levels of FITC-dextran and tight junction proteins (ZO-1, occludin, and claudin1) expression, H&E and AB-PAS stained tissue sections, and the mRNA expression levels of *MUC2* and *REG3G*. Compared with the diquat and 0.6-Se-FMT groups, 0.0-Se-FMT significantly increased the gut permeability of diquat-exposed mice, as indicated by the higher levels of serum FITC-dextran and the lower levels of tight junction proteins expression (Fig. 7c–e). Moreover, the FMT from the 0.6-Se group significantly increased the height of villi (Fig. 7f and Supplementary Fig. 7a), the number of goblet cells (Fig. 7g and Supplementary Fig. 7b), and the mRNA expression levels of *MUC2* and *REG3G* compared with the diquat model and 0.0-Se-FMT groups (Fig. 7h–i).

### Effects of FMT on antioxidant capacity and immune responses in mice exposed to diquat

**Fig. 8:**
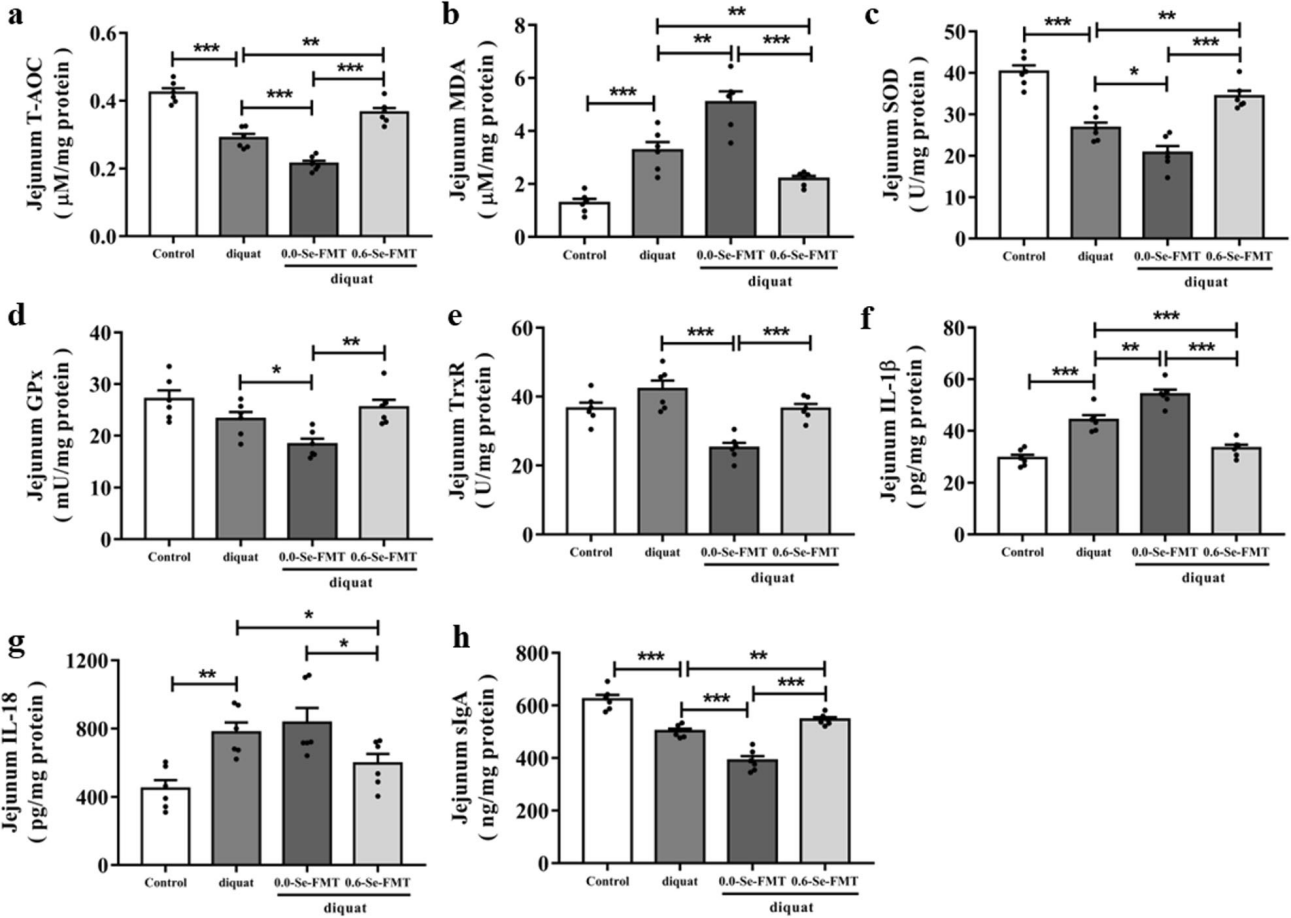
Effects of FMT on the antioxidant capacity and immune response in mice exposed to diquat. **a**–**e** Oxidative stress response markers including T-AOC (**a**), MDA (**b**), SOD (**c**), GPx (**d**), TrxR (**e**) (*n* = 6). **f** Level of IL-1β in the jejunum of mice (*n* = 6). **g** Level of IL-18 in the jejunum of mice (*n* = 6). **h** Level of sIgA in the jejunum of mice (*n* = 6). Data are expressed as mean ± SEM. ^*^P < 0.05; ^**^P < 0.01; ^***^P < 0.001.

As shown in Fig. 8a–e, there was a significant decrease in T-AOC and SOD levels and an increase in the MDA level in diquat-exposed mice, which were strongly inhibited by 0.6-Se-FMT. However, 0.0-Se-FMT led to a significant decrease in the level of T-AOC and an increase in the level of MDA compared with the diquat-induced model group. In the diquat-induced model group, the 0.0-Se-FMT significantly reduced their activity, while 0.6-Se-FMT pretreatment increased their activity. In addition, increased levels of IL-1β and IL-18 and decreased levels of sIgA were observed in mice exposed to diquat compared with the normal control group. However, pretreatment with 0.6-Se-FMT significantly inhibited the increase of the IL-1β level and decrease of sIgA level compared to the diquat-induced model group. Remarkably, 0.0-Se-FMT aggravated the increase of pro-inflammatory cytokines and the decrease of sIgA induced by diquat (Fig. 8f–h).

### Effects of FMT on gut microbiota in mice exposed to diquat

**Fig. 9:**
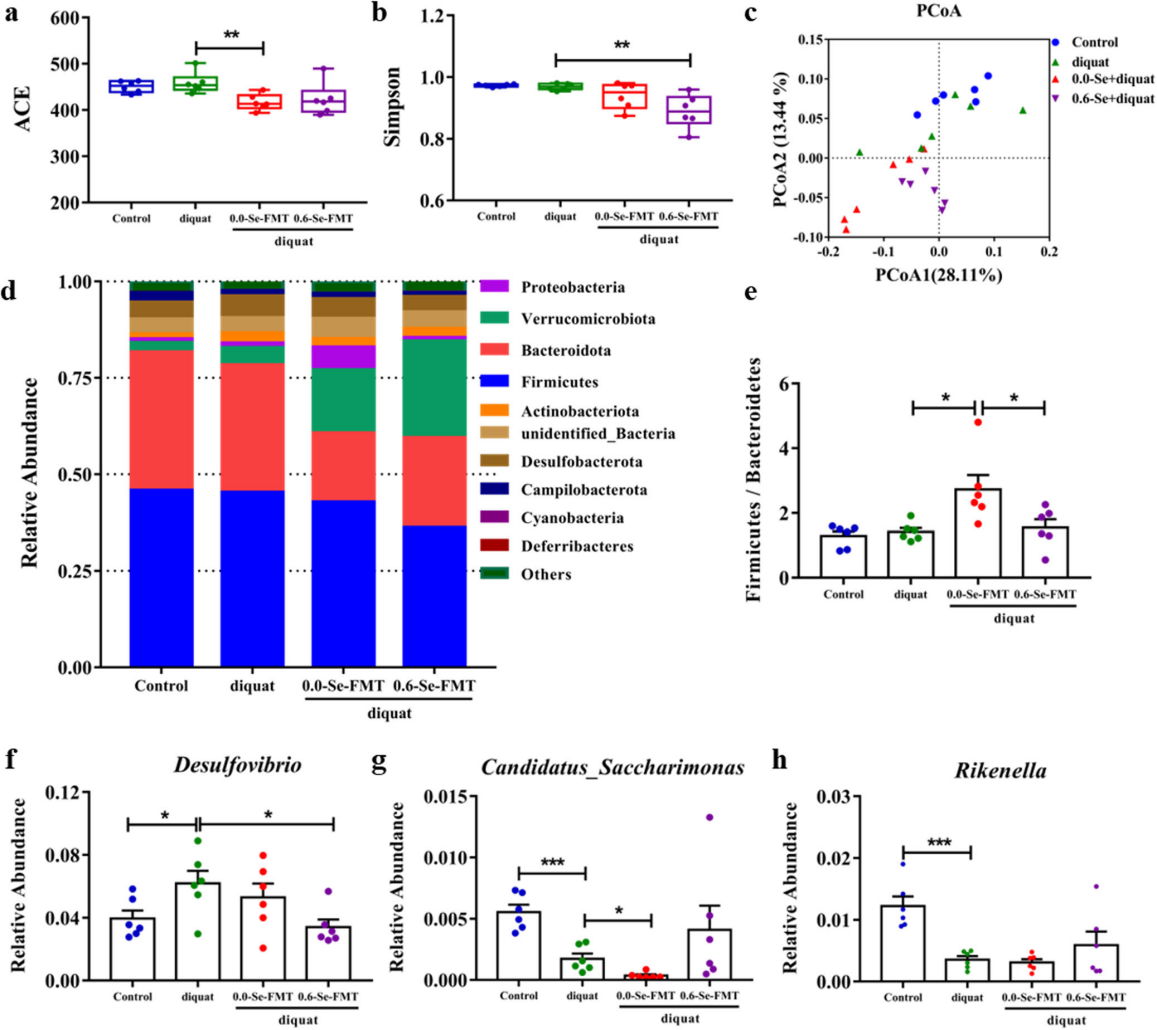
Effects of FMT on gut microbiota in mice exposed to diquat. **a**, **b** Alpha diversity index (*n* = 6). **c** PCoA (*n* = 6). **d** The relative abundance composition of gut microbiota at the phylum level (*n* = 6). **e** Ratio of *Firmicutes* to *Bacteroides* (*n* = 6). **f**–**h** Differences in gut microbiota between the genus level (*n* = 6). Data are expressed as mean ± SEM. ^*^P < 0.05; ^**^P < 0.01; ^***^P < 0.001.

16S rDNA sequencing was used to analyze the changes caused by FMT in the gut microbiota at Se concentrations from 0.0-Se and 0.6-Se. Compared with the diquat-induced model group, the ACE index of the 0.0-Se-FMT group decreased significantly and the Simpson index of the 0.6-Se-FMT group decreased significantly (Fig. 9a and b). Further PCoA analysis revealed that the gut microbiota of mice in different groups had obvious clustering, indicating that FMT and/or diquat significantly changed the composition of the gut microbiota (Fig. 9c). At the phylum level, 0.0-Se-FMT significantly increased the F/B ratio compared with the diquat model and 0.6-Se-FMT groups, which means that the gut microbiota of this group is disordered (Fig. 9d and e). At the genus level, compared with the normal control group, diquat exposure led to significant increases in the levels of *Desulfovibrio* and decreases in the level of *Candidatus_Saccharimonas* and *Rikenella*. In addition, 0.0-Se-FMT further reduced the abundance of *Candidatus_Saccharimonas*, and 0.6-Se-FMT decreased the abundance of *Desulfovibrio* and increased the abundance of *Candidatus_Saccharimonas* and *Rikenella* compared with the diquat exposure model group (Fig. 9f–h).

### FMT from 0.6-Se activated the Nrf2 signaling pathway to inhibit diquat-induced intestinal mitochondrial dysfunction

**Fig. 10:**
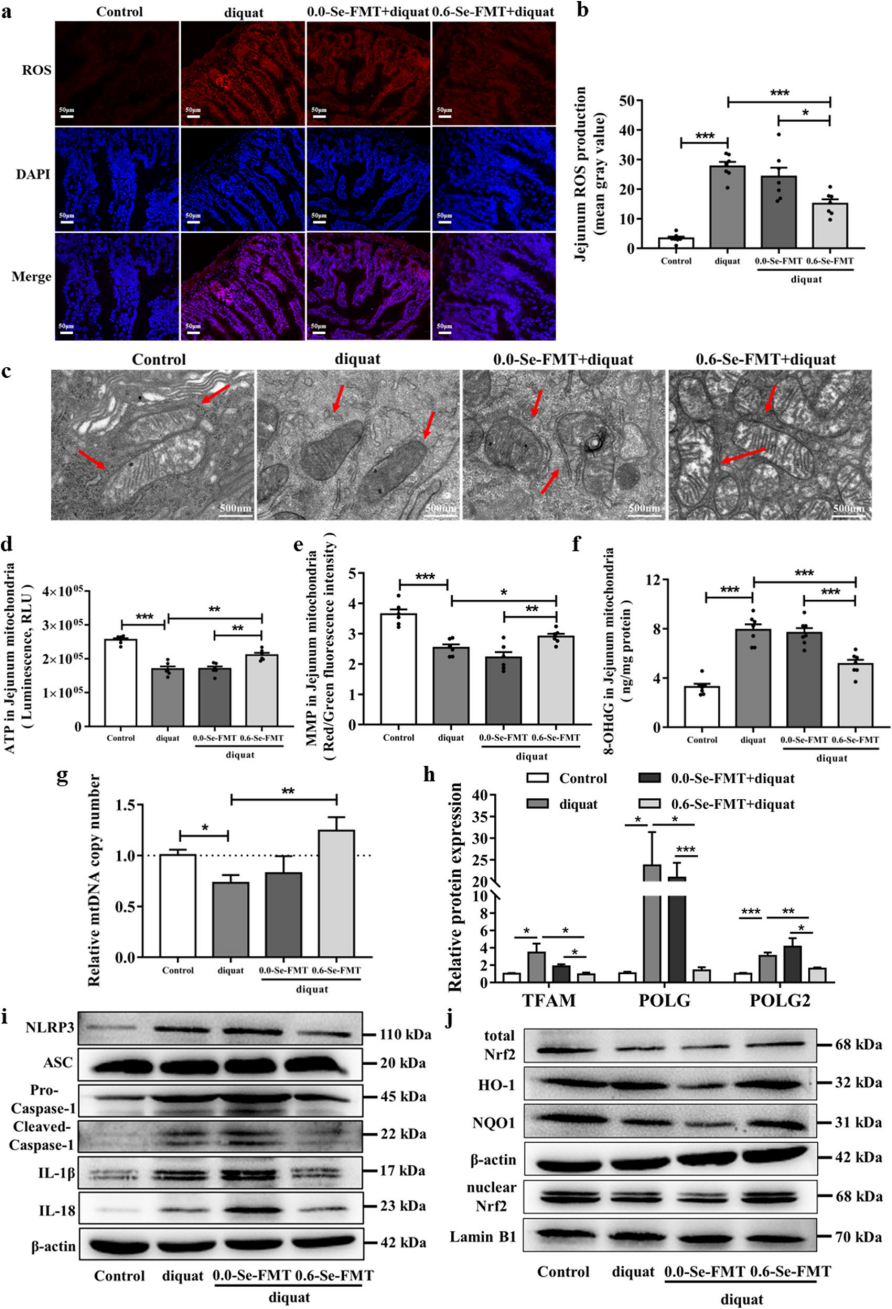
FMT from the 0.6-Se group activated the Nrf2 signaling pathway to inhibit the diquat-induced intestinal mitochondrial dysfunction. **a**, **b** ROS production was determined by DHE staining (*n* = 7). **c** Mitochondrial ultrastructure was observed by TEM. **d** Mitochondrial ATP level in the jejunal mitochondria (*n* = 6). **e** MMP was measured by JC-1 staining in the jejunal mitochondria (*n* = 6). **f** Level of 8-OHdG in the jejunal mitochondria (*n* = 6). **g** The mtDNA copy number was analyzed by qPCR (*n* = 6). **h** The mRNA expression levels of *TFAM*, *POLG,* and *POLG2* in the jejunum (*n* = 6). **i** The expression levels of NLRP3 inflammatory protein were measured by Western blot analysis (*n* = 3). **j** Nrf2 activation and expression levels of its downstream proteins measured by Western blot analysis (*n* = 3). Data are expressed as the fold change versus the control group (set to 1). Data are expressed as mean ± SEM. ^*^P < 0.05; ^**^P < 0.01; ^***^P < 0.001.

As shown in Fig. 10a and b, treatment with diquat obviously induced the overproduction of ROS in the jejunum, which was clearly abolished by FMT from the 0.6-Se pretreatment. Representative images of mitochondrial ultrastructure from each group are shown in Fig. 10c, which reveal that the 0.6-Se-FMT can protect the mitochondrial morphology from diquat-induced oxidative stress damage. In addition, compared with the normal control group, the ATP content and MMP level were significantly decreased in the jejunal mitochondria from mice of the diquat exposure model group. However, the 0.6-Se-FMT effectively alleviated diquat-induced intestinal mitochondrial dysfunction (Fig. 10d and e). In addition, the level of 8-OHdG in the 0.6-Se-FMT group was significantly lower than that in the diquat exposure model and 0.0-Se-FMT groups (Fig. 10f). The mtDNA copy number can be used as an important feature of mitochondrial function. Therefore, the relative level of mtDNA copy number was measured by qPCR analysis. As shown in Fig. 10g, pretreatment with the 0.6-Se-FMT significantly alleviated the reduction in the mtDNA copy number in mice exposed to diquat. The mtDNA copy number is correlated to the expression of mitochondrial biogenesis genes. Notably, the expression of *TFAM*, *POLG,* and *POLG2* in the diquat model exposure group was higher than that in the normal control group and pretreatment with the 0.6-Se-FMT significantly down-regulated the mRNA levels of TFAM, POLG, and POLG2 in the jejunum when compared with the group treated with diquat alone (Fig. 10h). We also examined whether NLRP3 and Nrf2 are involved in the antioxidative effect of FMT from the 0.6-Se. Western blot analysis showed that diquat exposure can lead to an increase in the level of the NLRP3 inflammasome and subsequent IL-1β and IL-18 expression, and FMT from 0.0-Se further increased their expression. However, pretreatment with the 0.6-Se-FMT significantly inhibited the activation of the NLRP3 inflammasome compared to the diquat exposure model group and 0.0-Se-FMT group (Fig. 10i and Supplementary Fig. 8a). Furthermore, the expression of Nrf2 (total Nrf2 and nuclear Nrf2), NQO-1, and HO-1 in the jejunum from mice in the diquat exposure model group decreased significantly compared with the normal control group, and the FMT from 0.0-Se further decreased their expression. However, the 0.6-Se-FMT effectively improved the expression level of Nrf2 and downstream antioxidant proteins compared with the diquat exposure model and 0.0-Se-FMT groups, indicating that FMT from the 0.6-Se activated the Nrf2 signaling pathway to inhibit the diquat-induced intestinal mitochondrial dysfunction (Fig. 10j and Supplementary Fig. 8b).

## Discussion

The micronutrient Se is incorporated, through the selenocysteine biosynthesis pathway into the redox-active amino acid selenocysteine, which is translationally incorporated into antioxidant selenoproteins in which Se acts as a cofactor^25^. Endemic Se deficiency is a worldwide nutritional problem and the imbalance of Se intake is associated with many human diseases (e.g., Keshan disease, Kashin-Beck disease, Thyroid autoimmune disease, Prostate cancer risk, and type 2 diabetes)^26^. Se deficiency in the body, results in decreased content and activity of selenoproteins, leading to decreased antioxidant capacity and immune response of the body and increased oxidative damage, as confirmed by our previous findings^27^. Thus, Se supplementation is a top priority to address the problem of Se deficiency, especially in those regions of the world where it is pervasive. However, the bioavailability and adverse side effects of Se are closely related to its chemical forms. As therapeutic agents for nutritional supplements and diseases, there are different forms of Se, including inorganic and organic Se compounds and SeNPs, which have excellent biological activities, low toxicity and comparable bioavailability^28^. The results of the safety evaluation based on cell and animal models have indicated that the order of toxicity of the Se species was as follows: selenate > selenite > selenomethionine > SeNPs^22,29,30^. Based on the excellent antioxidant activity of SeNPs^31^, SeNPs have been investigated for the treatment of various diseases, such as diabetes, Alzheimer’s disease, and inflammation-related diseases, such as rheumatoid arthritis^32^.

The effects of Se on the integrity of the intestinal barrier have recently received increasing attention. In fact, several large-scale human studies indicated that Se deficiency could increase the incidence of IBDs and colorectal cancer^33,34^. In this study, we found that a Se-deficient diet increased intestinal permeability decreased the antioxidant capacity, and increased the levels of pro-inflammatory cytokines, thus making mice more vulnerable to diquat-induced oxidative stress. In the intestine, *MUC2* is the most abundant mucus protein secreted by goblet cells. *MUC2* expression is essential for disease prevention, as shown by studies indicating that *MUC2* knockout mice will spontaneously develop colitis^12^. In addition, *REG3G* is closely related to the function of the intestinal barrier. Lack of *REG3G* will increase the number of mucosa-associated bacteria, leading to intestinal barrier dysfunction^35^. Our results showed that Se deficiency resulted in the reduction of *REG3G* expression levels in the jejunum. Dietary SeNP supplementation upregulated the expression of *MUC2* and *REG3G* in the jejunum, in a dose-dependent manner in mice exposed to diquat. Oxidative stress and overproduction of ROS have been identified to be among the major causes of intestinal barrier dysfunction. Due to continuous exposure to exogenous factors, the intestine becomes very susceptible to oxidative stress, so the intestine is the central organ of oxidative stress^36^. Structural and functional impairment of mitochondria caused by any adverse conditions can induce increased production of ROS. An excessive accumulation of ROS initiates a series of oxidative damage reactions in the cell, and becomes itself the primary target of its own attack to cause further mitochondrial structural damage and dysfunction^13^. In this study, we found that dietary SeNPs supplementation protected intestinal mitochondria against ROS attack in mice exposed to diquat. The NLRP3 inflammasome plays a key role in the processing and release of inflammatory cytokines, such as IL-1β and IL-18. Accumulating evidence suggests that mitochondria are common mediators of the NLRP3 inflammasome activation induced by a wide range of inflammatory stimuli^37^. Our results showed that Se deficiency can lead to an increase in the level of the NLRP3 inflammasome and subsequent IL-1β and IL-18 expression, and diquat exposure further increased its expression. These findings indicate that the NLRP3 inflammasome senses mitochondrial dysfunction and may explain the frequent association of mitochondrial damage with inflammatory diseases^38^. Moreover, it has been fully demonstrated that the redox homeostasis-associated transcription factor, Nrf2, has a potential role in the activation of NLRP3 inflammasomes^39^. Indeed, previous studies have confirmed that Nrf2 is an important transcription factor that regulates the cellular oxidative stress response, and is also a central regulator that maintains intracellular redox homeostasis^40^. In this study, dietary SeNPs supplementation upregulated the expression of Nrf2 and downstream antioxidant proteins in a dose-dependent manner. In addition, the levels of the NLRP3 inflammasome and subsequent IL-1β and IL-18 expression showed a decreasing trend along with the increasing levels of dietary Se in the jejunum of mice exposed to diquat. Together, these findings suggested that dietary SeNPs supplementation activated the Nrf2 pathway to inhibit ROS-induced NLRP3 inflammasome activation in mice after exposure to diquat.

Growing evidence has demonstrated the critical role of the gut microbiota in the physiological and pathological conditions of the host and also helps repair the intestinal mucosal barrier damage^41^. Dietary Se supplementation affects the composition and abundance of the gut microbiota, and the gut microbiota will also affect the bioavailability of Se and the expression of selenoproteins in mice^24^. Similarly, dietary SeNPs supplementation significantly altered the diversity of the gut microbiota of mice in this study. *Firmicutes* (F) and *Bacteroidetes* (B) are two dominant phyla representing together up to 90% of the total gut microbiota. The F/B ratio is known to be associated with different pathological conditions including intestinal barrier dysfunction^42^. A decreasing trend of the F/B ratio was observed in the 0.6-Se group compared with the 0.0-Se group. At the genus level, significant increases in the levels of *Bacteroides* and *Clostridium_XlVa* and decreases in the level of *Desulfovibrio* were found in mice fed with 0.6-Se compared with the results for mice given the 0.0-Se diet. *Bacteroides* is a prominent member of the intestinal microbiota and are good at breaking down carbohydrates and producing propionic acid^43^. Recent studies have shown that *Bacteroides thetaiotaomicron* plays an important role in nutrient absorption and promoting barrier function through its influence on goblet cell development and mucus secretion^44^. *Clostridium_XlVa* is reported to be a common bacterial strain capable of degrading carbohydrates into hydrogen and producing volatile fatty acids, such as acetate and butyrate, which could be useful in treating diseases, such as IBD^45,46^. *Desulfovibrio* is a conditional pathogen to produce LPS endotoxin, its relative abundance and related LPS level are higher in the intestinal tract of obese and UC patients than in healthy individuals. Moreover, a high level of *Desulfovibrio* will damage intestinal epithelial cells and the intestinal barrier integrity^47,48^. As a metabolite of the gut microbiota, similar alterations in the levels of SCFAs have also been observed in the cecal contents of mice fed a diet supplemented with different concentrations of SeNPs. Compared with the 0.0-Se group, supranutritional Se increased the content of total SCFAs, butyrate, isobutyrate, valerate, and isovalerate. Such disturbances resulting from a Se-deficient diet may impair normal nutrient intake and microbiota functions, while changes in microbiota composition may, in turn, significantly contribute to intestinal-related dysfunction and various pathological conditions. Callejon-Leblic et al.^49^ found that Se-supplementation modulated the concentration of GPx and selenoalbumin as well as the metal homeostasis, being influenced by microbiota disruption, which suggested an intertwined mechanism. Further study showed an important effect of Se-supplementation on gut microbiota-depleted (Abx) mice metabolism^50^. This provided evidence for a key interaction between selenium intake-microbiome-metabolism, but further studies are needed to elucidate the specific mechanisms.

Recent evidence suggests that there is an interaction between mitochondria and gut microbiota. The gut microbiota has been shown to regulate key transcriptional co-activators, transcription factors, and enzymes involved in mitochondrial biogenesis. Moreover, the gut microbiota and its metabolites, such as SCFAs and secondary bile acids, also contribute to host energy production, modulation of ROS production, and inflammation in the gut by attenuating TNF-α-mediated immune response and induction of inflammasomes, such as NLRP3. On the other hand, mitochondrial processes, particularly mitochondrial ROS production, have a key role in regulating the gut microbiota by modulating intestinal barrier function and mucosal immune responses^51,52^. This study found that Se deficiency resulted in a gut microbiota phenotype that is more susceptible to diquat-induced intestinal barrier dysfunction and the FMT from the 0.6-Se group activated the Nrf2 signaling pathway to prevent diquat-induced intestinal mitochondrial dysfunction.

Our results showed that deficient Se supplementation resulted in a gut microbiota phenotype that was more susceptible to diquat-induced intestinal barrier dysfunction and supernutritional SeNPs supplementation resulted in a gut microbiota phenotype that prevents the oxidative stress-induced intestinal barrier dysfunction. Overall, biogenic SeNPs exhibit attractive antioxidant activity, which may be mainly manifested in the following aspects: (1) improve antioxidant defense capacity and reduced inflammation; (2) improved bioenergetics; (3) modulation of the gut microbial composition and metabolism. In summary, Se deficiency induced a redox imbalance and a gut microbiota phenotype that is more susceptible to diquat-induced intestinal barrier dysfunction through the Nrf2-mediated NLRP3 signaling pathway in mice exposed to diquat. Dietary supplementation with biogenic SeNPs synthesized by *L. casei* ATCC 393 effectively alleviated diquat-induced intestinal barrier dysfunction by enhancing the antioxidant capacity, preventing the overproduction of ROS, improving mitochondrial structure and function, regulating the immune response, and maintaining intestinal microecological homeostasis by regulating Nrf2-mediated NLRP3 signaling pathway.

## Methods

### Reagents

Biogenic SeNPs synthesized by *L. casei* ATCC 393 were prepared according to our previously established methods^22^. Diquat (Cat# DRE-CA12960000) was obtained from Dr Ehrenstorfer GmbH (Wesel, Germany). RNAex pro reagent (Cat# AG21102), evo M-MLV mix kit with gDNA clean (Cat# AG11728) and SYBR^®^ green premix pro taq HS qPCR kit (Cat# AG11701) were purchased from Accurate Biology (Hunan, China). FITC-dextran (Cat# FD4) was obtained from Sigma-Aldrich (Saint Louis, MO, USA). DAO assay kit (Cat# A088-2-1), D-LA ELISA kit (Cat# H263-1-2), TrxR activity assay kit (Cat# A119-1-1), GPx activity assay kit (Cat# A005-1-2), SOD assay kit (Cat# A001-3-2), T-AOC assay kit (Cat# A015-2-1) and MDA assay kit (Cat# A003-1-2) were purchased from Nanjing Jiancheng Bioengineering Institute (Nanjing, China). ELISA kits for IL-1β (Cat# JL18442), IL-18 (Cat# JL20253), sIgA (Cat# JL11763) and 8-OHdG (Cat# JL12294) were purchased from Jianglaibio Company (Shanghai, China). MMP assay kit (Cat# C2006), tissue mitochondria isolation kit (Cat# C3606) and ATP assay kit (Cat# S0026) were purchased from Beyotime Biotechnology (Shanghai, China). Primary antibodies for Nrf2 (1:1000; Cat# A0674), NQO-1 (1:1000; Cat# A19586), HO-1 (1:1000; Cat# A1346), claudin 1 (1:1000; Cat# A2196), occludin (1:1000; Cat# A2601), ZO-1 (1:500; Cat# A0659), NLRP3 (1:1000; Cat# A5652), caspase-1 (1:1000; Cat# A0964), apoptosis-associated speck-like protein containing a caspase recruitment domain (ASC; 1:1000; Cat# A16672), IL-1β (1:1000; Cat# A1112), IL-18 (1:1000; Cat# A1115), β-actin (1:15,000; Cat# AC026) and Lamin B1(1:1000; Cat# A1910), and secondary antibody for HPR goat anti-rabbit IgG (1:2000; Cat# AS014) were purchased from Abclone Biotechnology (Wuhan, China).

### Animals and experimental diets

The animal experiment was approved by the Institutional Animal Care and Use Committee of the Northwestern Polytechnical University (Xi’an, China) and conducted in accordance with the National Institutes of Health guidelines for the care and use of experimental animals. Male C57BL/6 mice were purchased from the Experimental Animal Center of Air Force Medical University (Xi’an, China). The experimental diets with different Se levels were obtained from Trophic Animal Feed High-tech Co., Ltd. (Suzhou, China). The Se-deficient diet (0.0-Se) contained Se at a level <0.01 mg/kg. The 0.0-Se diet was enriched with biogenic SeNPs synthesized by *L. casei* ATCC 393 at a final Se content of 0.3 mg/kg (0.3-Se) or 0.6 mg/kg (0.6-Se) for the Se supplied diets. The Se concentration in drinking water, mice diet and isotonic salt solution, where the Se concentrations in drinking water, normal saline, and PBS were all 0 mg/L (Detection limit not reached). In addition, the actual Se concentrations of the Se-deficient diet, the 0.3-Se diet, and 0.6-Se diet were 0.009, 0.320, and 0.603 mg/kg, respectively. The maintenance diet (Cat# 1010009, Se concentration: 0.25 mg/kg) for SPF grade mice was purchased from Xietong Pharmaceutical Bio-engineering Co., Ltd (Nanjing, China).

### Animal experiments

Animal Experiment I. A total of 72 male C57BL/6 mice aged 3 weeks (body weight: 12.35 ± 1.03 g) were randomly assigned to three groups with 24 mice per group. As shown in the scheme (Fig. 1a), the mice from each group had *ad libitum* to the above-mentioned 0.0-Se, 0.3-Se, and 0.6-Se diets, and were maintained for 10 weeks. Two weeks before the end of the experiment, each mouse was transferred into a sterilized cage and fecal samples were collected from the cages. The fecal pellets were used for future FMT treatments. Twelve hours before the end of the experiment, 12 randomly selected mice from different dietary supplementation of the SeNPs groups were administered 25 mg/kg of body weight diquat suspended in 100 μL of PBS by intraperitoneal injection. The other mice were intraperitoneally injected with 100 μL of PBS.

Animal Experiment II. After a week of adaptation, 40 six-week-old mice (body weight: 28.33 ± 1.42 g) were randomly assigned to four groups: normal control group (control), diquat-induced oxidative damage model group (diquat), 0.0-Se-FMT + diquat group and 0.6-Se-FMT + diquat group, with 10 mice in each group. First, the mice in the 0.0-Se-FMT + diquat group and 0.6-Se-FMT + diquat group were pretreated with a cocktail of antibiotics (50 μg/mL clindamycin, 50 μg/mL metronidazole, 50 μg/mL penicillin, 25 μg/mL vancomycin and/or 50 μg/mL neomycin) in the drinking water for 8 weeks. One day after antibiotic treatment, mice were gavage-fed for 7 weeks with the corresponding fecal transplant materials (200 μL) prepared from animal experiment I. Fecal transplantation materials were prepared as follows: 150 mg of the collected fecal samples were placed in 1 mL of normal saline, shaken well, and then centrifuged at 2500 rpm for 5 min at 4 °C for a total of two times. Subsequently, the supernatant was centrifuged at 8000 rpm for 5 min at 4 °C, and the pellet was resuspended in 1 mL of normal saline, mixed with 50% sterile glycerol to a final concentration of 20%, and then stored at −80 °C.

### Se content detection

The Se content in the organs, serum, and feces were measured by inductively coupled plasma-mass spectrometry (ICP-MS).

### mRNA expression analysis

Total RNA was extracted from liver and jejunum using the RNAex pro reagent. The quality and concentration of the total RNA was measured with an Implen nanophotometer (Munich, Germany). Then, cDNA was synthesized using an evo M-MLV mix kit with gDNA clean. Subsequently, real-time PCR was performed on a CFX96 Touch™ Real-Time PCR Detection System (Bio-Rad, USA) according to the SYBR^®^ green premix pro taq HS qPCR kit. The primers for target genes and a housekeeping gene (β-actin) are listed in Supplementary Table 1. The relative mRNA abundance of the selected genes was normalized to β-actin expression and was then calculated using the 2^−ΔΔCt^ method.

### Gut microbiota and metabolism analysis

The microbial community DNA was extracted using the MagPure Stool DNA KF kit B (Magen, China) following the manufacturer’s instructions. Variable regions V3–V4 of bacterial 16S rRNA gene was amplified with degenerate PCR primers, 341F (5′-ACTCCTACGGGAGGCAGCAG-3′) and 806R (5′- GGACTACHVGGGTWTCTAAT-3′). Gut microbiota composition was assessed using Illumina MiSeq platform and QIIME-based microbiota analysis (This method is described in detail in the Supplementary information).

The cecal contents were resuspended in a saturated NaCl solution. The samples were acidified with sulfuric acid (10%) and fatty acids were extracted with diethyl ether. The mixture was centrifuged at 12,000 rpm/min for 10 min and Na_2_SO_4_ was then added to the supernatant to remove the water content. The concentrations of short chain fatty acids (SCFAs) in the samples were analyzed by gas chromatography-mass spectrometry (GC-MS) on a TRACE1300-TSQ9000 Triple Quadrupole GC-MS/MS System (Thermo Fisher Scientific, Waltham, MA, USA).

### Biomarkers of intestinal barrier function damage

The alterations in intestinal permeability of the mice were determined using FITC-dextran (4 kDa). Four hours prior to the end of the experiment, mice were intragastrically administered with a dose of 400 mg/kg body weight of FITC-dextran and the concentration of FITC-dextran in the serum was measured with a fluorescence spectrophotometer. The levels of D-LA and DAO in the serum of mice were determined using corresponding kits according to the manufacturer’s instructions.

### Evaluation of intestinal morphology and goblet cells number in jejunum

The morphology and goblet cell number in the jejunum were evaluated by H&E staining and AB-PAS staining, respectively.

### Antioxidant capacity and immune responses detection

The T-AOC and MDA levels, and the SOD, GPx, and TrxR activities in the jejunum were determined using the corresponding kits according to the manufacturer’s instructions. ELISA kits were used to measure IL-1β, IL-18 and sIgA from serum and jejunum according to the manufacturer’s instructions.

### Detection of ROS generation in the jejunum

ROS generation in the jejunum was measured using dihydroethidine (DHE) staining.

### Ultrastructure of mitochondria and mitochondrial function detection

Jejunum were washed with PBS, then fixed with 2.5% glutaraldehyde overnight at 4 °C. After samples were processed according to the standard procedures, including staining, dehydration, embedding, and slicing into ultra-thin sections, the ultrastructure of mitochondria was observed on a Hitachi HT7800 transmission electron microscope (Hitachi Ltd., Tokyo, Japan). Mitochondria were isolated from fresh jejunum using the tissue mitochondria isolation kit. ATP, MMP and 8-OHdG levels in the jejunum mitochondria were determined using the corresponding kits.

### Mitochondrial DNA (mtDNA) in the jejunum

The relative mtDNA copy number was determined by calculating the DNA expression ratio of the mitochondrial cyclooxygenase 2 (*COX2*) gene and the nuclear Hexokinase 2 (*HK2*) gene. The primer sequences are listed in Supplementary Table 1. Data were analyzed by the 2^−ΔΔCt^ method.

### Western Blot Analysis

Total protein was isolated from jejunum using the RIPA buffer containing protease inhibitor cocktail (Topscience, Cat# C0001). Nuclear protein was isolated using a nuclear protein extraction kit. The protein concentration of the samples was measured by the BCA protein assay kit. Before loading, protein samples were boiled in the loading buffer, then samples of equal volume with equal amount of protein were loaded onto the SDS-PAGE gel, and then transferred to the PVDF membrane. The membranes were incubated overnight at 4 °C with the appropriate primary antibodies for ZO-1, occludin, claudin1, Nrf2, HO-1, NQO-1, NLRP3, IL-1β, IL-18, caspase-1, ASC, β-actin and Lamin-B1 followed by incubation for 1 h at room temperature with the appropriate secondary antibodies. Immunoreactive protein bands were visualized with the clarity Western ECL substrate kit (BioRad, Cat# 1705061) using Tanon 5200 Multi (Shanghai, China) and quantified using the Image J analyzer software (National Institute of Health, Bethesda, MD, USA). All blots or gels derive from the same experiment and that they were processed in parallel.

### Statistical analysis

The data are expressed as the mean ± SEM. Statistical analysis was performed using the GraphPad Prism 7.0 software (GraphPad Software Inc., San Diego, CA, USA). One-way analysis of variance (ANOVA) followed by a least significant difference multiple comparison test was used to determine the statistical significance for multiple comparisons, and Student’s *t*-test was used for the comparisons of two groups. *P* < 0.05 was considered as statistically significant.

## Supplementary information

Supplementary information

## Data Availability

The data that support the findings of this study are available from the corresponding author upon reasonable request. Raw sequence data that support the findings of this study have been deposited in the National Center for Biotechnology Information (NCBI) Sequence Read Archive (SRA) and are accessible through the accession numbers PRJNA777712 and PRJNA834901.
