# Supplementary material for: Dietary supplementation with biogenic selenium nanoparticles alleviate oxidative stress-induced intestinal barrier dysfunction
Source: NPJ Sci Food. 2022 Jun 23;6:30. doi: 10.1038/s41538-022-00145-3 (PMC9226128; doi:10.1038/s41538-022-00145-3)
Supplement: Supplementary file 1 — Supplementary information [file 41538_2022_145_MOESM1_ESM.pdf]

Supplementary information

Dietary supplementation with biogenic selenium nanoparticles alleviate oxidative stress-induced intestinal barrier dysfunction

Lei Qiao[1], Xinyi Zhang[1], Shanyao Pi[1], Jiajing Chang[1], Xina Dou[1], Shuqi Yan[1], Xiaofan Song[1], Yue Chen[1], Xiaonan Zeng[1], Lixu Zhu[1], Chunlan Xu[1*]

[1]The Key Laboratory for Space Bioscience and Biotechnology, School of Life Sciences, Northwestern Polytechnical University, Xi'an, Shaanxi, 710072, China

∗ Corresponding author: Chunlan Xu, Associate Professor, School of Life Sciences, Northwestern Polytechnical University, 127 Youyixi Road Xi'an, Shaanxi, 710072, China. E-mail: clxu@nwpu.edu.cn Telephone: +86-29-88460543, Fax: +86-29-88460332

Table of Contents

1 Supplementary Methods 3

2 Supplementary Results 4

2.1 Effects of different dietary SeNPs supplementation on Se content of mice 4

2.2 Relative mRNA expression levels of selenoproteins in the liver and jejunum 5

2.3 Effects of different dietary SeNPs supplementation on the intestinal barrier in mice exposed to diquat 6

2.4 Effects of different dietary SeNPs supplementation on immune responses in diquat-challenged mice 7

2.5 Effects of different dietary SeNPs supplementation on gut microbiota 8

2.6 Differences in fecal microbiota function prediction 9

2.7 Effects of FMT on the intestinal barrier in mice exposed to diquat 10

2.8 FMT from the 0.6-Se activated the Nrf2 signaling pathway to inhibit diquat-induced NLRP3 inflammatory activation 11

3 Supplementary Table 12

1 Supplementary Methods

Sequencing and bioinformatics analysis

Raw reads were filtered to remove adaptors and low-quality and ambiguous bases, and then paired-end reads were added to tags by the Fast Length Adjustment of Short reads program (FLASH, v1.2.11) to get the tags. The tags were clustered into OTUs with a cutoff value of 97% using UPARSE software (v7 .0.1090) and chimera sequences were compared with the Gold database using UCHIME (v4.2.40) to detect. Then, OTU representative sequences were taxonomically classified using Ribosomal Database Project (RDP) Classifier v.2.2 with a minimum confidence threshold of 0.6, and trained on the Greengenes database v201305 by QIIME v1.8.0. The USEARCH_global was used to compare all Tags back to OTU to get the OTU abundance statistics table of each sample.

Alpha and beta diversity were estimated by MOTHUR (v1.31.2) and QIIME (v1.8.0) at the OTU level, respectively. Sample cluster was conducted by QIIME (v1.8.0) based on UPGMA. The Venn plots in OTUs or in taxa were plotted with R package "VennDiagram" version 3.1.1. Species accumulation curves was plotted with R package version 3.1.1. Principal component analysis (PCA) in OTUs was plotted with R package "ade4". KEGG functions were predicted using the PICRUSt software. Barplot and heatmap of different classification levels was plotted with R package v3.4.1 and R package "gplots", respectively.

2 Supplementary Results

2.1 Effects of different dietary SeNPs supplementation on Se content of mice

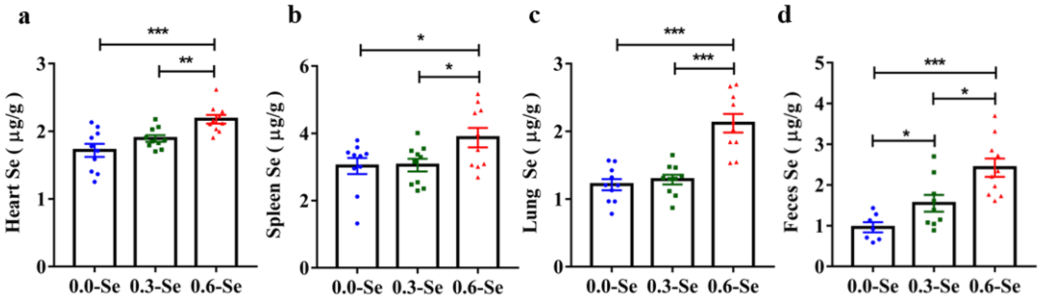

Supplementary Figure 1. Effects of different dietary SeNPs supplementation on Se content of mice. (a) The Se content in the heart (n=10). (b) The Se content in the spleen (n=10). (c) The Se content in the lung (n=10). (d) The Se content in the feces (n=10). Data are expressed as the mean ± S.E.M. $^{*}P < 0.05$; $^{**}P < 0.01$; $^{***}P < 0.001$.

2.2 Relative mRNA expression levels of selenoproteins in the liver and jejunum

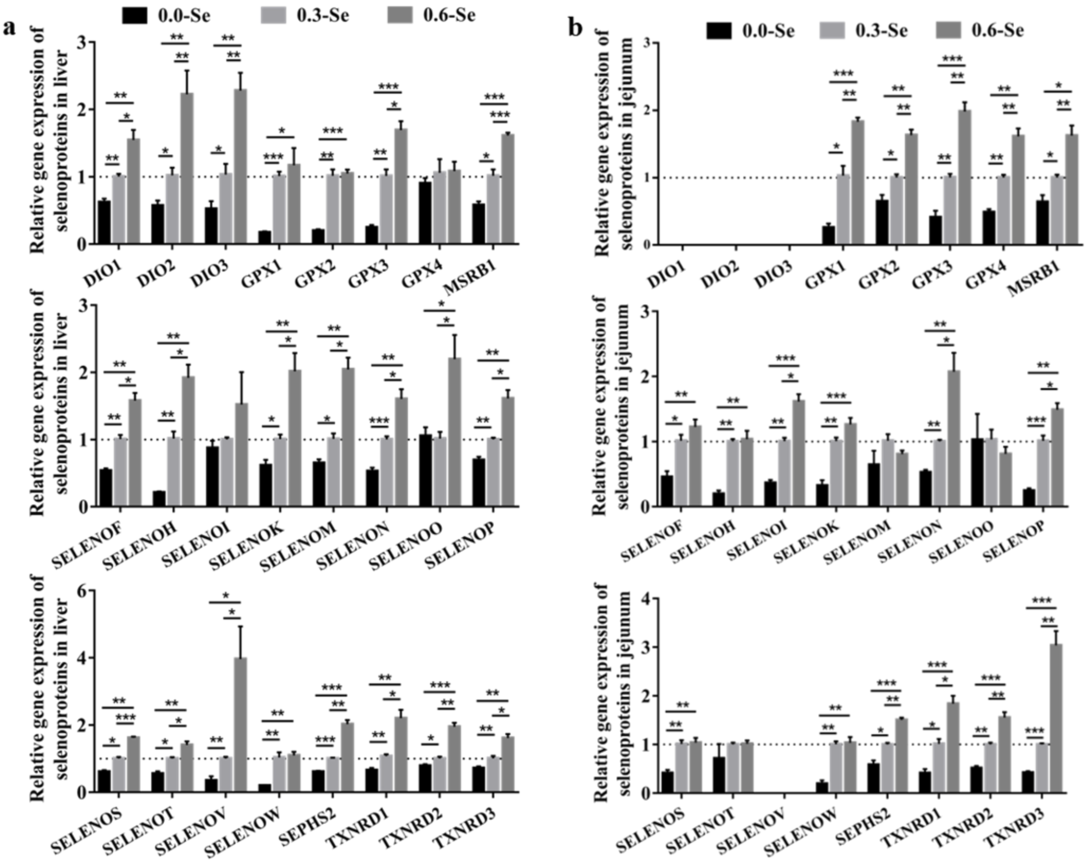

Supplementary Figure 2. (a) Relative mRNA expression levels of selenoproteins in liver (n=4). (b) Relative gene expression of selenoproteins in the jejunum (n=4). Data are expressed as the mean ± S.E.M. $^{*}P < 0.05$; $^{**}P < 0.01$; $^{***}P < 0.001$.

2.3 Effects of different dietary SeNPs supplementation on the intestinal barrier in mice exposed to diquat

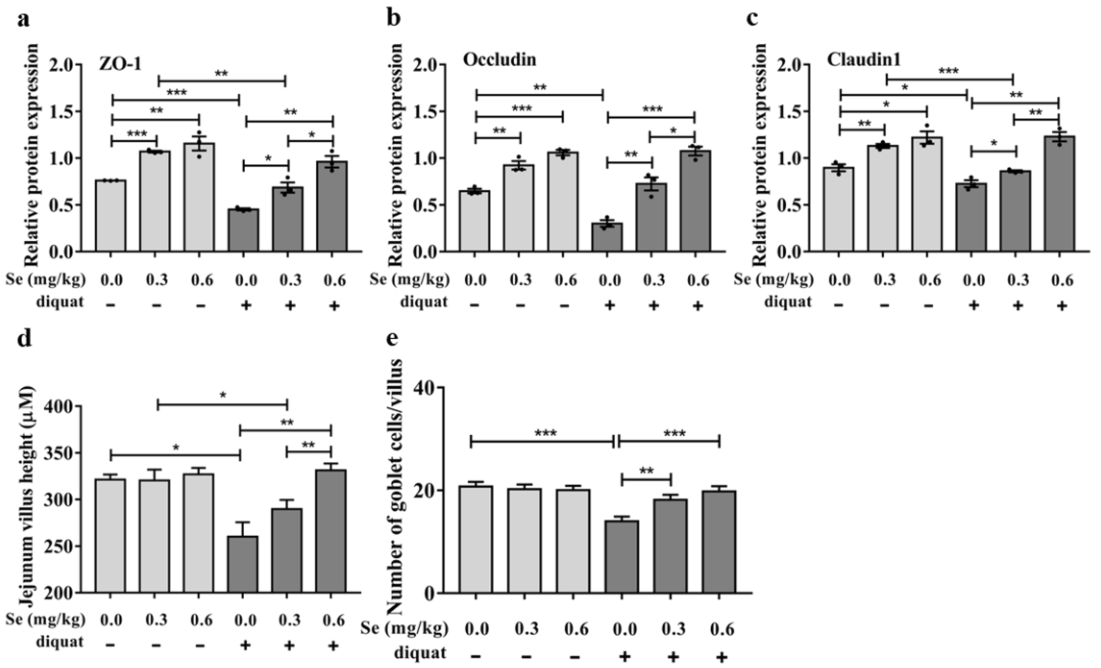

Supplementary Figure 3. Effects of different dietary SeNPs supplementation on the intestinal barrier in mice exposed to diquat. (a-c) Quantitative analysis of the protein expression levels of ZO-1 (a), occluding (b) and claudin-1(c) (n=3). (d) Quantification of jejunal villi height. (e) Quantification of jejunal goblet cells numbers. Data are expressed as the mean ± S.E.M. $^{*}P < 0.05$; $^{**}P < 0.01$; $^{***}P < 0.001$.

2.4 Effects of different dietary SeNPs supplementation on immune responses in diquat-challenged mice

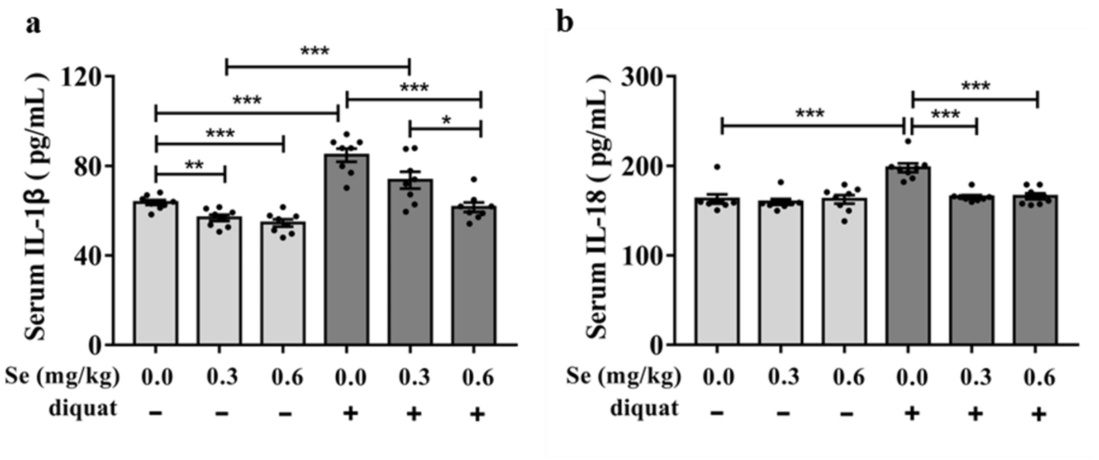

Supplementary Figure 4. Effects of different dietary SeNPs supplementation on immune responses in diquat-challenged mice. (a) Levels of IL-1β in the serum of mice (n=8). (b) Levels of IL-18 in the serum of mice (n=8). Data are expressed as the mean ± S.E.M. $^{*}P < 0.05$; $^{**}P < 0.01$; $^{***}P < 0.001$.

2.5 Effects of different dietary SeNPs supplementation on gut microbiota

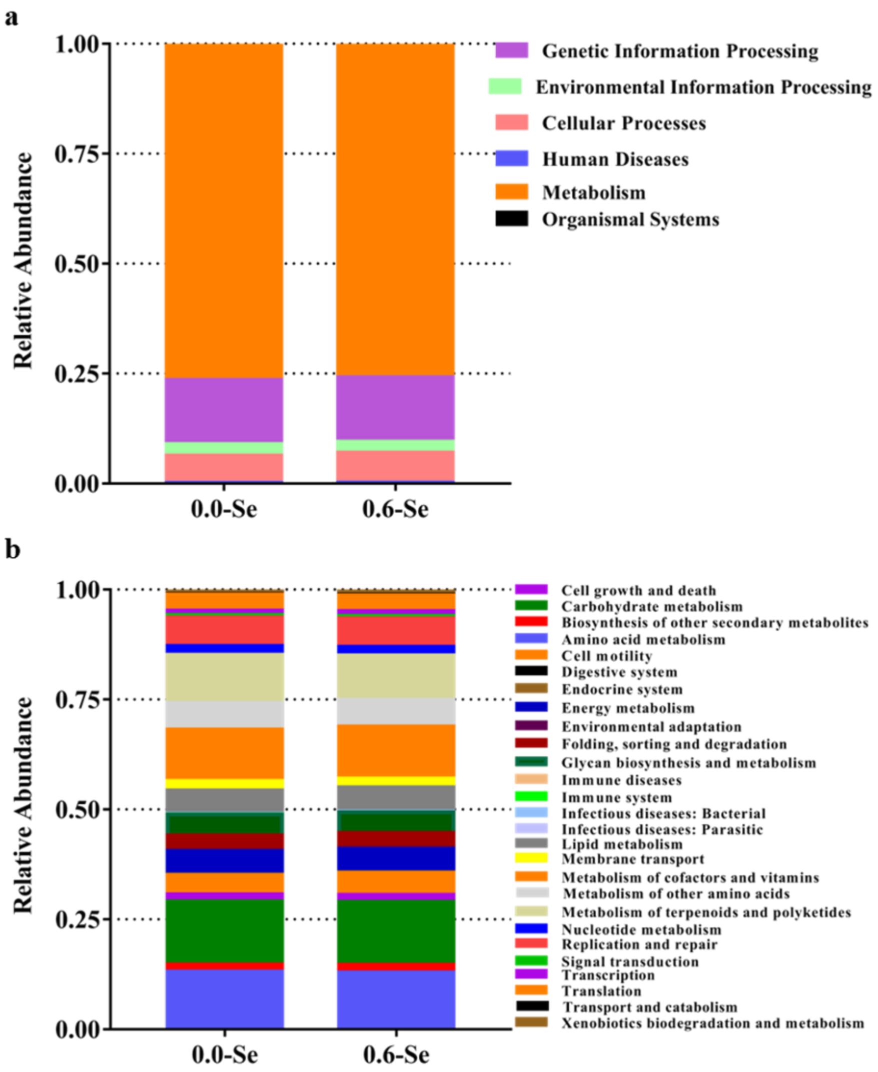

Supplementary Figure 5. Effects of different dietary SeNPs supplementation on gut microbiota. (a) Feces microbiota function prediction at the level 1. (b) Feces microbiota function prediction at the level 2.

2.6 Differences in fecal microbiota function prediction

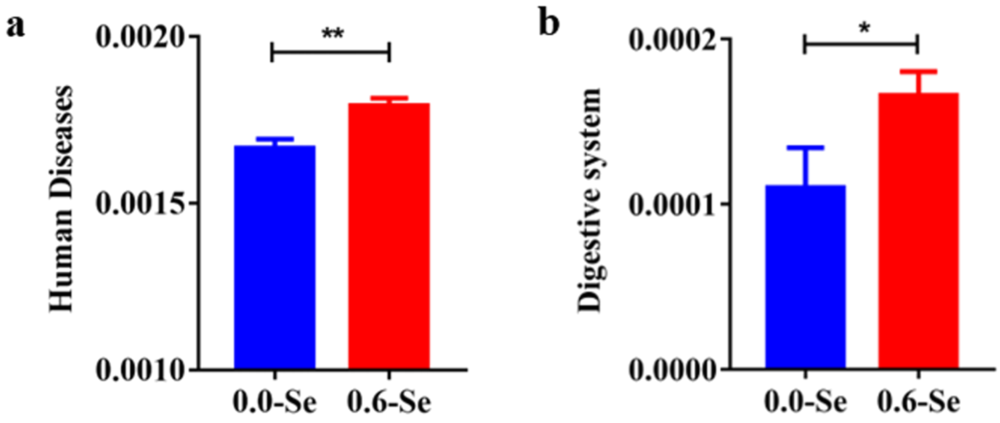

Supplementary Figure 6. (a) Differences in fecal microbiota function prediction at the KEGG level 1 ($n \geq 7$). (b) Differences in fecal microbiota function prediction at the KEGG level 2 ($n \geq 7$). $^{*}P < 0.05$; $^{**}P < 0.01$.

2.7 Effects of FMT on the intestinal barrier in mice exposed to diquat

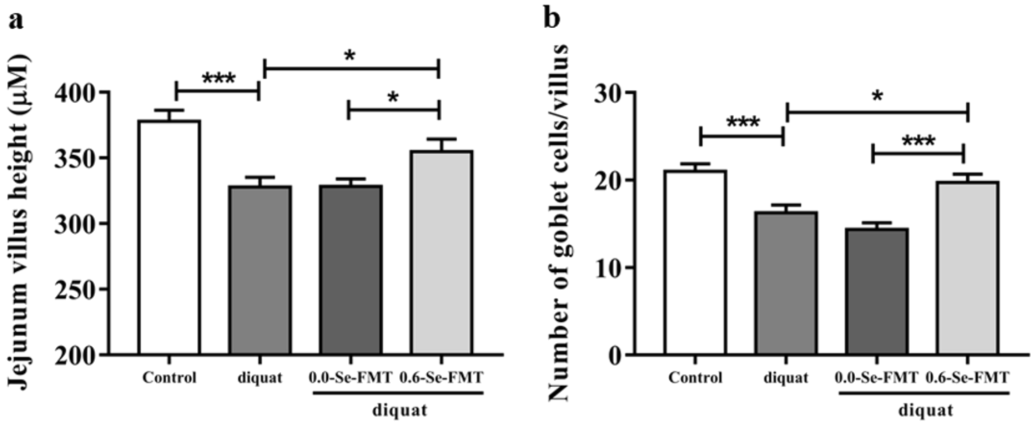

Supplementary Figure 7. Effects of FMT on the intestinal barrier in mice exposed to diquat. (a) Quantification of jejunal villi height. (b) Quantification of jejunal goblet cells numbers. Data are expressed as the mean ± S.E.M. $^{*}P < 0.05$; $^{***}P < 0.001$.

2.8 FMT from the 0.6-Se activated the Nrf2 signaling pathway to inhibit diquat-induced NLRP3 inflammatory activation

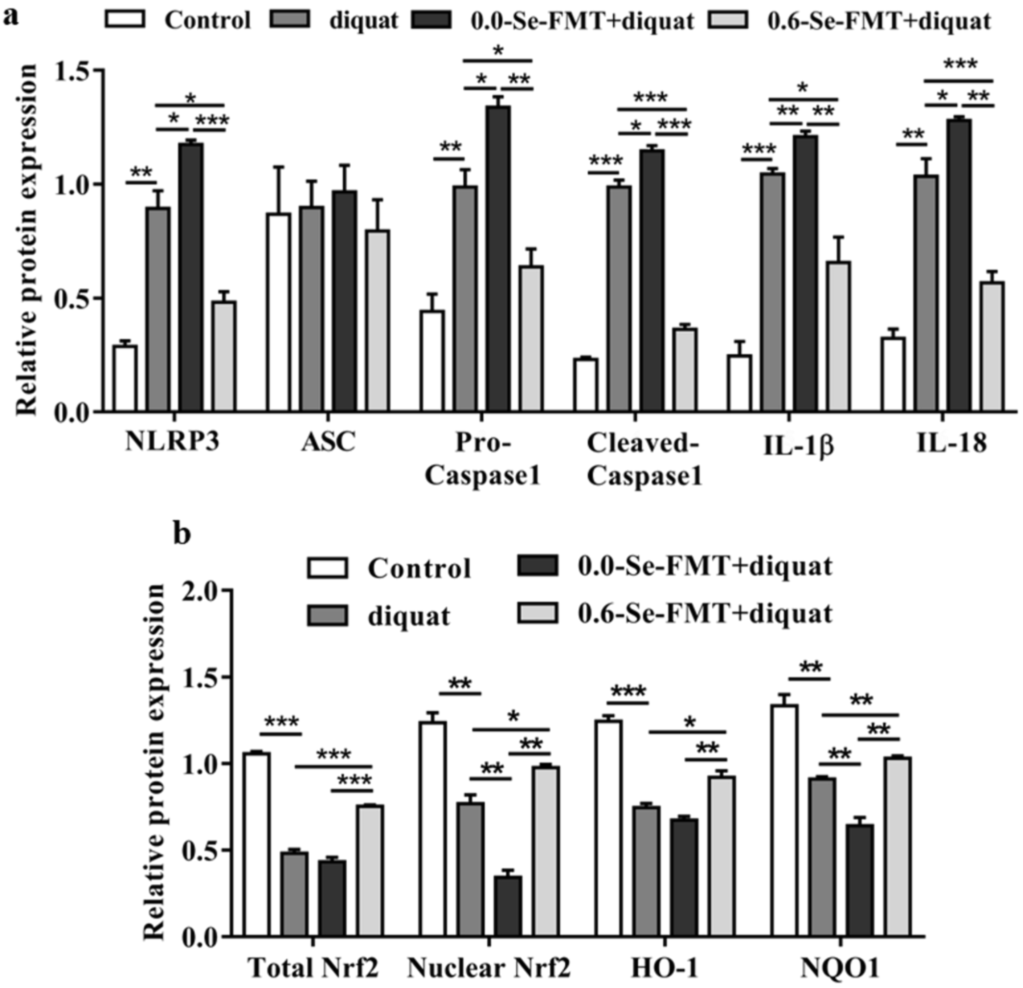

Supplementary Figure 8. FMT from the 0.6-Se activated the Nrf2 signaling pathway to inhibit diquat-induced NLRP3 inflammatory activation. (a) Quantitative analysis of the protein expression levels of NLRP3, ASC, pro-caspase-1, cleaved-caspase-1, IL-1β and IL-18. (b) Quantitative analysis of the protein expression levels of total Nrf2, nuclear Nrf2, HO-1 and NQO-1. Data are expressed as the mean ± S.E.M. $^{*}P < 0.05$; $^{**}P < 0.01$; $^{***}P < 0.001$.

3 Supplementary Table

Supplementary Table 1. The primers and sequences of qPCR

**Genes**	**Sequences (5'-3')**	**Accession number**
Selenoprotein genes		
DIO1	F: AAGCAAGAGGCAGGCATGTT<br>R: CGGCCAGAAAAGTGTTTCCA	NM_007860.3
DIO2	F: GGAATGTTGGCCAGTTTTGTTT<br>R: TGGTTACATGGGCTGGTGAGT	NM _010050.2
DIO3	F: AGGTGTCTGAGTTGCGCACTT<br>R: TGGCCTAGTACCATGCAACTGT	NM_172119.2
GPX1	F: AGGCTCACCCGCTCTTTACC<br>R: GGGTCGTCACTGGGTGTTG	NM_008160.6
GPX2	F: TGTGACGTCAATGGGCAGAA<br>R: AGGGCAGCTTGTCTTTCAGGTA	NM_030677.2
GPX3	F: ACAGGAGCCAGGCGAGAA<br>R: CCACCTGGTCGAACATACTTGA	NM_008161.3
GPX4	F: GCCGGCTACAACGTCAAGTT<br>R: GGCATCGTCCCCATTTACAC	NM_001037741.3
MSRB1	F: CAGCCTCAGTCACCGAATGA<br>R: ACCACCCTGGCTGGCATA	AF195142.1
SELENOF	F: TGGACGACAACGGGAACAT	NM_053102.2

	R: CCACACTGTCTGTGTTCCACTTG	
SELENOH	F: ATTCCCGGCTGCTGGTTT<br>R: GGCGCGTTGGTGGAATAA	NM_001033166.2
SELENOI	F: GCTTTGGGAGCAGTGTGCTAT<br>R: AGCTCTGCTCCAGCAAGATCA	NM_027652.2
SELENOK	F: GGGTAGGATCAGTCACCTTCGT<br>R: TTCCTCATCCACCAGCCATT	NM_019979.2
SELENOM	F: GGAGACCTGTGGAGGATGACA<br>R: TCGGTGACAAAGGCCTTCAC	NM_053267.2
SELENON	F: ACCGGATGGCCACCAGTT<br>R: GGTCAGCCGTTCAAGCTGTT	NM_029100.2
SELENOO	F: CCCCAGGTATGCAAGTGGAA<br>R: AGTGGCAGTTCAGGCTCCAA	NM_027905.2
SELENOP	F: CAGGGTCTGCAATTGCTTGA<br>R: GAAAAGCCCCTGTCAGCTACA	X99807.1
SELENOS	F: TGTTAAGCGGCAAGAGGCTTT<br>R: GGGCATTTAGATCTTCCTGCAT	NM_024439.3
SELENOT	F: TGCACTCGCATTCGTGACA<br>R: ACTGGAGCTCACCGCATTG	NM_001040396.2
SELENOV	F: GCTGCTAGCGCTCTCTTGAAG<br>R: GTGGATCGAGGGTTTCTGATTT	NM_175033.3
SELENOW	F: GCCGTTCGAGTCGTGTATTGT	AF015284

	R: TCTCCTTGAGCTGGAGGTACTTG	
SEPHS2	F: CGTTGGCATCGTGGAGAAG<br>R: CGCGAGGCTTGTCAATGAT	NM_009266.3
TXNRD1	F: CACAAACAGCGAGGAGACCAT<br>R: TTCCTACCGCCAGCAACACT	BC037643.1
TXNRD2	F: GTTCACGGTGGCGGATAGG<br>R: GCTCCCTCATGCATACCATCTT	NM_013711.3
TXNRD3	F: GGGACATACTGGACGGCAAA<br>R: TAGCAGCTTGCCTGCCTGTA	C076605.1
	Biomarker genes of the intestinal barrier function	
MUC2	F: CTGACCAAGAGCGAACACAA<br>R: CATGACTGGAAGCAACTGGA	NM_023566.4
Reg3g	F: TCAGGACATCTTGTGTCTGTGCTC<br>R: CATCCACCTCTGTTGGGTTCA	NM_011260.2
	Genes related to mitochondrial biosynthesis	
TFAM	F: GGAATGTGGAGCGTGCTAAAA<br>R: TGCTGGAAAAACACTTCGGAATA	NM_009360.4
POLG	F: AGAAGCAGAGCCTGCCTTAC<br>R: CTCTGCCAAGCAGACCTCC	NM_001360095.1
POLG2	F: CTTGCAAGACAGAGAGCCG<br>R: CTGGGTGTCTGATTGCTGTTC	NM_001353435.1
	mtDNA copy number	

COX2	F: GTTGATAACCGAGTCGTT<br>R: CCTGGGATGGCATCAGTT	NC_005089.1
Hexokinase2	F: GCCAGCCTCTCCTGATTTTAGTGT<br>R: GGGAACACAAAAGACCTCTTCTGG	NC_000072.7
	Housekeeping gene	
β-actin	F: TCACCCACACTGTGCCCATCTACGA<br>R: GGATGCCACAGGATTCCATACCCA	NM_007393.5
